# Pulmonary function in young childhood cancer survivors: results from a prospective multicentre cohort

**DOI:** 10.1183/23120541.00500-2026

**Published:** 2026-09-07

**Authors:** Maša Žarković, Christina Schindera, Nicolas Waespe, Daniel Trachsel, Anne Mornand, Marc Ansari, Philipp Latzin, Claudia E. Kuehni

**Affiliations:** 1 Childhood Cancer Research Group, Institute of Social and Preventive Medicine, University of Bern, Bern, Switzerland; 2 Graduate School for Health Sciences, University of Bern, Bern, Switzerland; 3 Division of Paediatric Oncology/Haematology, University Children's Hospital Basel, University of Basel, Basel, Switzerland; 4 Division of Pediatric Hematology and Oncology, Department of Pediatrics, Inselspital, Bern University Hospital, University of Bern, Bern, Switzerland; 5 CANSEARCH Research Platform for Pediatric Oncology and Hematology, Department of Pediatrics, Gynecology and Obstetrics, Faculty of Medicine, University of Geneva, Geneva, Switzerland; 6 Pediatric Intensive Care and Pulmonology, University Children's Hospital Basel, Basel, Switzerland; 7 Division of Pediatric Pulmonology, Department of Women, Child and Adolescent, University Hospital of Geneva, Geneva, Switzerland; 8 Division of Pediatric Oncology and Hematology, Department of Women, Child and Adolescent, University Hospital of Geneva, Geneva, Switzerland; 9 Division of Paediatric Respiratory Medicine and Allergology, Department of Paediatrics, Inselspital, University Hospital, University of Bern, Bern, Switzerland

## Abstract

**Background:**

Childhood cancer survivors (CCSs) are at risk of pulmonary late effects, but post-treatment lung function remains understudied. Current guidelines recommend screening only for symptomatic survivors treated with lung-damaging treatments. We evaluated pulmonary function, risk factors and respiratory symptoms in a representative paediatric CCS cohort, including those with standard treatments.

**Methods:**

In this prospective multicentre study, we included CCSs aged 6–21 years, stratified as high-risk (thoracic radiotherapy/surgery, busulfan/bleomycin/nitrosourea chemotherapy and haematopoietic stem cell transplantation (HSCT)) or standard-risk (other systemic treatment). Pulmonary function was assessed *via* spirometry (forced expiratory volume in 1 s (FEV_1_) and forced vital capacity (FVC)), body plethysmography (total lung capacity (TLC)) and diffusing capacity for carbon monoxide (*D*_LCO_), expressed as z-scores using Global Lung Initiative references. We assessed respiratory symptoms *via* questionnaires and analysed treatment associations with pulmonary function using multivariable linear regression.

**Results:**

With a response rate of 90%, 251 CCSs participated (median 7 years post-diagnosis). Mean z-scores for FEV_1_, FVC, TLC and *D*_LCO_ were lower in high-risk (−0.70, 95% confidence interval −0.96 to −0.43; −0.91, −1.16 to −0.66; −0.54, −0.78 to −0.31; and −0.17, −0.55 to 0.22, respectively) than standard-risk survivors (−0.10, −0.26 to 0.06; −0.22, −0.37 to −0.06; −0.17, −0.33 to −0.01; and 0.32, 0.15 to 0.49). Thoracic surgery and nitrosoureas were associated with lower TLC (−0.53 and −1.37), HSCT with reduced FEV_1_ and FVC (−0.80 and −0.84) and thoracic radiotherapy with lower *D*_LCO_ (−0.63). Respiratory symptoms were reported by 32%, but 64% of those with impaired lung function were asymptomatic.

**Conclusion:**

Pulmonary function was mostly normal in standard-risk CCSs, whereas high-risk survivors showed mild reductions, often asymptomatic. These findings support targeted surveillance based on treatment exposure rather than symptoms to guide long-term care.

## Shareable abstract

**Lung damage can go unnoticed in childhood cancer survivors. This study highlights who needs risk-based screening.** https://bit.ly/3RbUjSU

## Introduction

Survival after childhood cancer has markedly improved in recent decades, with over 80% of patients in high-income countries now expected to become long-term survivors [1–3]. However, this success has brought an increasing burden of late treatment-related complications [4]. Pulmonary morbidity is among the leading causes of non-malignant mortality in childhood cancer survivors (CCSs) [5]. Several cancer treatments have been found to adversely affect the lungs, including chemotherapeutic agents such as bleomycin, busulfan and nitrosoureas, thoracic surgery, chest radiotherapy and haematopoietic stem cell transplantation (HSCT). Damage to the lung parenchyma, vasculature or airways [6, 7], may result in restrictive, obstructive or diffusion impairments on pulmonary function tests (PFTs), with cumulative incidence increasing over time [4].

We recently led the development of the International Late Effects of Childhood Cancer Guideline Harmonisation Group (IGHG) recommendations for pulmonary surveillance in CCSs, which were based on a systematic review of the literature [8]. This process identified considerable methodological weaknesses in existing studies, including predominantly retrospective designs, non-standardised PFT assessments, and inconsistent use of Global Lung Initiative (GLI)-based reference values. Many studies focused on selected subgroups, such as HSCT recipients [9–11] or those treated with thoracic radiotherapy [12, 13], whereas others included only adult survivors [14–16], leaving important gaps in early post-treatment data and risk factor assessment in childhood and adolescence [17]. Evidence was insufficient to assess pulmonary outcomes in survivors treated with other systemic therapies not previously established as lung-damaging, and to evaluate how respiratory symptoms relate to PFT impairments. As a result, the IGHG could only recommend routine PFTs for symptomatic survivors exposed to known lung-damaging treatments. Therefore, we conducted a prospective cohort study of paediatric CCSs with diverse treatment exposures. We aimed to determine the prevalence of pulmonary function abnormalities using standardised PFTs, evaluate associations with clinical and treatment-related factors, and assess the frequency of respiratory symptoms in relation to PFT impairments.

## Methods

### Study design and procedure

The Swiss Childhood Cancer Survivor Study FollowUp–Pulmo (SCCSS-FU Pulmo) is a prospective cohort evaluating long-term pulmonary health in CCSs. It is embedded in routine follow-up care at the University Children's Hospitals of Bern, Basel and Geneva, and linked to the Childhood Cancer Registry (ChCR), a nationwide registry that captures over 95% of children diagnosed with cancer [18] according to the International Classification of Childhood Cancer (ICCC-3) [19].

To ensure near-complete recruitment, eligible survivors were identified through a two-way approach: local study teams regularly screened upcoming follow-up appointments and cross-checked against lists from the ChCR to identify eligible patients. The treating physicians approved and scheduled PFTs as part of routine oncological care. Patients received an information letter, consent form and respiratory questionnaire before the visit. If consent and questionnaire were not returned before or during the visit, up to two reminders were sent to allow inclusion of data. Detailed procedures have been published [20]. The study was approved by the Ethics Committee of the Canton of Bern (KEK-BE: 2019-00739). For the present analysis, pulmonary outcomes were assessed cross-sectionally using the PFTs performed at the clinical visit during which participants were recruited.

### Study population

We included all CCS aged 6 to 21 years who completed treatment, were in remission and still in follow-up care at participating centres between June 2022 and July 2025. Survivors treated only with surgery or radiotherapy outside the thorax were excluded due to minimal expected impact on pulmonary function.

Participants were classified into two risk groups based on treatment exposure. High-risk survivors received therapies previously associated with impaired pulmonary outcomes, including pulmotoxic chemotherapy (busulfan, bleomycin, carmustine or lomustine), pulmotoxic radiotherapy (radiation to the chest (mantle, mediastinal or whole lung fields), abdomen (whole or any upper field) or total body irradiation), HSCT or thoracic surgery (procedures involving the lungs or chest wall such as lobectomy, wedge resection or thoracotomy) [6, 21]. Standard-risk survivors were treated with any other systemic anticancer therapy (chemotherapy, immunotherapy or targeted agents).

In a secondary step, we further subdivided the high-risk group by thoracic surgery to distinguish the potential effects of surgical interventions from those of other high-risk treatments.

### Pulmonary function tests

Pulmonary function assessment included spirometry, body plethysmography and diffusing capacity of the lung for carbon monoxide (*D*_LCO_), performed according to European Respiratory Society/American Thoracic Society (ERS/ATS) standards [22]. Tests were conducted in paediatric lung function laboratories by trained technicians with on-site quality control, and results were validated by paediatric pulmonologists. Pulmonary function outcomes were grouped into three physiological domains: markers of restriction (forced vital capacity (FVC), total lung capacity (TLC) and functional residual capacity (FRC)); markers of obstruction (forced expiratory volume in 1 s (FEV_1_), FEV_1_/FVC ratio and forced mid-expiratory flow (FEF_25–75%_)); and markers of diffusion capacity (*D*_LCO_ and carbon monoxide transfer coefficient (*K*_CO_)). Spirometry was performed pre-bronchodilation, and *D*_LCO_ values were corrected for haemoglobin concentration if a measurement was available within 30 days. Outcomes were expressed as sex-, age-, height- and ethnicity-adjusted z-scores using GLI reference equations, calculated with the official GLI calculator (https://gli-calculator.ersnet.org/) [23–26]. The lower limit of normal (LLN) was defined as a z-score <−1.645, and impairments further classified by severity: mild (z-score −1.645 to −2.5), moderate (−2.5 to −4) and severe (<−4) [22]. Further details on parameters and reference standards are provided in supplementary tables S1 and S2.

### Respiratory symptoms

At the time of pulmonary function assessment, participants completed a questionnaire assessing respiratory symptoms in the past 12 months. Questionnaires were parent-completed for children <14 years and self-completed by older participants. The questionnaire included items on respiratory symptoms at rest and during exertion (wheeze, dyspnoea and cough) and chronic cough (>4 weeks). English translations of original German/French questions are provided in supplementary table S1.

### Clinical and demographic characteristics

We extracted clinical and demographic information from the medical records of participating centres. This included sex, age at study, age at diagnosis, time since diagnosis, cancer treatments and anthropometric measures (height, weight and body mass index (BMI)), converted into z-scores using World Health Organisation references [27]. Current asthma was defined based on either a documented diagnosis in medical records or self-report in the questionnaire. Smoking exposure was assessed *via* questionnaire: active smoking in participants ≥14 years, and passive smoking as parental smoking for participants <14 years or any self-reported exposure in those ≥14 years (supplementary table S1).

### Statistical analysis

We summarised continuous variables using means with 95% confidence intervals or medians with interquartile ranges (IQRs), and categorical variables as frequencies with percentages. In the first analytical step, we compared PFT outcomes between the standard-risk and high-risk groups using independent two-sample t-tests, after checking distributional assumptions, and we additionally estimated between-group differences with 95% CIs. In a second step, we further stratified the high-risk group by thoracic surgery and compared PFT outcomes between the standard-risk group and the two high-risk subgroups using t-tests to assess whether results (*e.g.* restriction) were explained by thoracic surgery alone. Categorical variables were compared using chi-squared or Fisher's exact test.

In addition to comparing risk groups, we also examined associations with individual treatment exposures using multivariable linear regression models. Exposures were selected *a priori* based on clinical relevance and previous evidence of pulmonary effects. For each physiological domain, we used one key outcome as the dependent variable: FEV_1_/FVC z-score (obstruction), TLC z-score (restriction) and *D*_LCO_ z-score (diffusion). Exposure variables included pulmotoxic chemotherapy (bleomycin, busulfan, nitrosoureas), thoracic surgery, pulmotoxic radiotherapy and HSCT. All models were adjusted for age at study, sex and time since diagnosis. In sensitivity analyses, we additionally adjusted for asthma, to account for potential confounding by pre-existing or concurrent respiratory disease, and for study centre to assess whether differences between centres affected the results. We also considered smoking exposure and BMI in sensitivity analyses. We used Stata v.16.1 (StataCorp) and R v.4.4.2 (R Foundation for Statistical Computing).

## Results

### Characteristics of the study population

Of 278 eligible survivors identified, 251 participated (response rate 90%) (supplementary figure S1). The median age at study was 14 years (IQR 10–17), and median time since diagnosis was 7 years (IQR 4–9) (table 1). The most common cancer diagnoses were leukaemia (49%), lymphoma (12%) and neuroblastoma (9%). Almost all participants (99%) received chemotherapy, including 28 (11%) with pulmotoxic chemotherapy. 35 (14%) received pulmotoxic radiotherapy, 20 (8%) underwent thoracic surgery and 33 (13%) HSCT.

**TABLE 1:** Characteristics of participating childhood cancer survivors, overall and stratified by treatment-related risk groups

	Total	Standard risk	High risk	p*-*value^#^
	N=251, 100%	n=181, 72%	n=70, 28%	
**Demographic characteristics**				
Sex				0.649
Male	142 (57)	104 (57)	38 (54)	
Female	109 (43)	77 (43)	32 (46)	
Age at study, years	14 (10–17)	14 (10–17)	14 (10–16)	0.841
Age at diagnosis, years	5 (3–10)	5 (3–10)	5 (2–10)	0.965
Time since diagnosis, years	7 (4–9)	7 (4–9)	7 (4–9)	0.791
**Clinical characteristics**				
Weight z-score at study	0.1 (−0.5–0.9)	0.3 (−0.3–1.1)	−0.3 (−1.0–0.5)	<0.001
Height z-score at study	0.1 (−0.8–0.6)	0.1 (−0.7–0.7)	−0.1 (−1.2–0.6)	0.024
BMI z-score at study	0.3 (−0.4–1.3)	0.7 (−0.3–1.4)	−0.2 (−1.0–0.7)	<0.001
Cancer diagnosis (ICCC-3 main group)				<0.001
Leukaemia	124 (49)	105 (58)	19 (27)	
Lymphoma	30 (12)	24 (13)	6 (9)	
CNS neoplasms	14 (6)	6 (3)	8 (11)	
Neuroblastoma	22 (9)	10 (6)	12 (17)	
Renal tumours	20 (8)	7 (4)	13 (19)	
Hepatic tumours	3 (1)	3 (2)	0 (0)	
Malignant bone tumours	10 (4)	6 (3)	4 (6)	
Soft tissue sarcomas	16 (6)	12 (7)	4 (6)	
Germ cell tumours	4 (2)	2 (1)	2 (3)	
Other neoplasms^¶^	2 (1)	1 (<1)	1 (1)	
LCH	6 (2)	5 (3)	1 (1)	
History of relapse	21 (8)	6 (3)	15 (21)	<0.001
Any chemotherapy	248 (99)	181 (100)	67 (96)	0.126
Pulmotoxic chemotherapy	28 (11)	0 (0)	28 (40)	<0.001
Bleomycin	2 (<1)	0 (0)	2 (3)	
Busulfan	18 (7)	0 (0)	18 (26)	
Carmustine	1 (<1)	0 (0)	1 (1)	
Lomustine	7 (3)	0 (0)	7 (10)	
Thoracic surgery^+^	20 (8)	0 (0)	20 (29)	<0.001
Pulmotoxic radiotherapy	35 (14)	0 (0)	35 (50)	<0.001
Mediastinal/lung radiation^§^	14 (6)	0 (0)	14 (19)	
Abdominal radiation^§^	18 (7)	0 (0)	18 (25)	
Total body irradiation	9 (4)	0 (0)	9 (13)	
HSCT	33 (13)	0 (0)	33 (45)	<0.001
Autologous	15 (6)	0 (0)	15 (21)	
Allogeneic	18 (7)	0 (0)	18 (25)	
Asthma	16 (6)	12 (7)	4 (6)	0.710
Passive smoking^ƒ^	72 (32)	48 (29)	24 (39)	0.127
Active smoking^##^	12 (10)	10 (12)	2 (6)	0.298

Data are presented as n (%) and median (IQR). BMI: body mass index; ICCC-3: International Classification of Childhood Cancer, Third Edition; LCH: Langerhans cell histiocytosis; CNS: central nervous system; IQR: interquartile range; HSCT: haematopoietic stem cell transplantation. ^#^: p-values comparing high-risk and standard-risk patients calculated from chi-squared or Fisher's exact tests for categorical variables and from t-tests for continuous variables. ^¶^: other malignant epithelial neoplasms, malignant melanomas, and unspecified malignant neoplasms. ^+^: thoracoscopic resection (n=4), wedge resection or metastasectomy of the lung (n=6), open tumour resection including mediastinal or chest wall tumours (n=8), complex cardiac or vascular surgery (n=1), combined thoracoscopic and open tumour resection (n=1). ^§^: six participants received both mediastinal/lung and abdominal radiotherapy. ^ƒ^: passive smoking indicates either having a parent who is an active smoker (reported by parents of participants younger than 14 years) or reported by adolescents (participants older than 14) as being exposed to passive smoking of any source. ^##^: active smoking was asked only in adolescents (older than 14 years), total n=112.

Standard-risk survivors accounted for 72% of the cohort, while 28% were classified as high-risk. High-risk survivors were shorter (median z-score −0.1 *versus* 0.1), slimmer (−0.3 *versus* 0.3) and had lower BMI z-scores (−0.2 *versus* 0.7) than standard-risk. Cancer types differed by risk group, reflecting distinct treatment protocols. A current asthma diagnosis was found in 16 (6%) and 12 (10%) of those aged ≥14 years reported active smoking.

### Pulmonary function parameters

PFTs were successful in most of the 251 participants: 95% had valid spirometry, 86% body plethysmography and 79% *D*_LCO_, with lower completion rates for the latter two due to limited cooperation in younger children. Overall, test completion and quality were comparable across centres (supplementary figure S1 and table 2).

**TABLE 2:** Pulmonary function parameters among childhood cancer survivors, reported overall and stratified by treatment-related risk groups. Parameters include measures of restriction, obstruction, and diffusion capacity, with prevalence of impairment and group comparisons

	Total	Standard risk	High risk	p*-*value^#^	Effect estimates (95% CI)^¶^
	N=251 (100%)	n=181 (72%)	n=70 (28%)		
**Restriction^+^**					
FVC	238 (95)	175 (97)	63 (90)		
Mean (95% CI)	3.18 (3.02–3.34)	3.29 (3.09–3.49)	2.87 (2.60–3.14)	0.025	−0.42 (−0.79– −0.05)
z-score	−0.40 (−0.54– −0.26)	−0.22 (−0.37– −0.06)	−0.91 (−1.16– −0.66)	<0.001	−0.69 (−0.99– −0.39)
z-score<LLN	30 (13, 8–17)	16 (9, 5–13)	14 (22, 12–33)	0.007	13 (2–24)
TLC	216 (86)	156 (86)	60 (86)		
Mean (95% CI)	4.35 (4.14–4.55)	4.50 (4.25–4.75)	3.94 (3.61–4.27)	0.015	−0.56 (−1.01– −0.11)
z-score	−0.27 (−0.41– −0.14)	−0.17 (−0.33– −0.01)	−0.54 (−0.78– −0.31)	0.014	−0.37 (−0.66– −0.08)
z-score<LLN	16 (8, 4–11)	9 (6, 2–9)	7 (12, 4–20)	0.138	6 (−3–15)
FRC	216 (86)	156 (86)	60 (86)		
Mean (95% CI)	2.25 (2.14–2.37)	2.31 (2.17–2.45)	2.11 (1.93–2.30)	0.125	−0.20 (−0.45–0.06)
z-score,	0.01 (−0.11–0.13)	0.01 (−0.14–0.15)	−0.02 (−0.19–0.23)	0.913	0.01 (−0.25–0.28)
z-score<LLN	7 (3, 1–7)	7 (4, 2–9)	0 (0)	0.704	1 (−5–7)
**Obstruction^+^**					
FEV_1_	238 (95)	175 (97)	63 (90)		
Mean (95% CI)	2.79 (2.66–2.92)	2.87 (2.71–3.03)	2.55 (2.33–2.78)	0.035	−0.32 (−0.62– −0.02)
z-score	−0.26 (−0.40– −0.12)	−0.10 (−0.26–0.06)	−0.70 (−0.96– −0.43)	<0.001	−0.60 (−0.91– −0.29)
z-score<LLN	23 (10, 6–13)	10 (6, 2–9)	13 (21, 10–31)	0.001	15 (4–25)
FEV_1_/FVC	238 (95)	175 (97)	63 (90)		
Mean (95% CI)	0.89 (0.88–0.90)	0.89 (0.88–0.90)	0.90 (0.88–0.92)	0.164	0.01 (−0.01–0.03)
z-score	0.28 (0.15–0.42)	0.24 (0.07–0.40)	0.41 (0.16–0.66)	0.271	0.17 (−0.14–0.48)
z-score<LLN	13 (6, 3–8)	12 (7, 3–11)	1 (2, 0–5)	0.193	−5 (−10– −0.4)
FEF_25–75%_	238 (95)	175 (97)	63 (90)		
Mean (95% CI)	3.30 (3.15–3.46)	3.35 (3.16–3.53)	3.18 (2.90–3.45)	0.345	−0.17 (−0.52–0.18)
z-score,	−0.09 (−0.23–0.05)	−0.08 (−0.24–0.08)	−0.12 (−0.41–0.16)	0.772	−0.05 (−0.37–0.27)
z-score<LLN	20 (8, 5–12)	14 (8, 4–12)	6 (10, 2–17)	0.683	2 (−7–10)
**Diffusion impairment^+^**					
*D*_LCO_ corrected for Hb	198 (79)	143 (80)	55 (77)		
Mean (95% CI)	7.78 (7.39–8.16)	8.07 (7.62–8.52)	7.03 (6.31–7.74)	0.016	−1.04 (−1.89– −0.20)
z-score	0.16 (−0.02–0.34)	0.32 (0.15–0.49)	−0.17 (−0.55–0.22)	0.008	−0.49 (−0.85– −0.13)
z-score<LLN	13 (7, 3–10)	6 (4, 1–7)	7 (13, 3–22)	0.027	9 (−0.8–18)
*K*_CO_ corrected for Hb	198 (79)	143 (80)	55 (77)		
Mean (95% CI)	1.92 (1.87–1.97)	1.90 (1.85–1.95)	1.98 (1.86–2.11)	0.124	0.08 (−0.0–0.19)
z-score	0.52 (0.35–0.68)	0.44 (0.28–0.61)	0.72 (0.29–1.14)	0.145	0.19 (−0.17–0.55)
z-score<LLN	3 (2, 0–3)	2 (1, 0–3)	1 (2, 0–5)	0.829	0.4 (−4–4)
**Any impairment^§^**					
FVC, TLC, FRC, FEV_1_, FEV_1_/FVC, FEF_25–75%_, *D*_LCO_ or *K*_CO_ z-score<−1.645	71 (28)	46 (25)	25 (36)	0.104	
Obstructive, restrictive, mixed, or diffusion capacity impairment^ƒ^	38 (15)	24 (13)	14 (20)	0.182	

Data are presented as n (%), mean (95% CI) or n (%, 95% CI). *D*_LCO_: diffusing capacity of the lungs for carbon monoxide; FEF_25–75%_: forced expiratory flow at 25% to 75% of FVC; FEV_1_: forced expiratory volume in 1 s; FRC: functional residual capacity; FVC: forced vital capacity; Hb: haemoglobin; *K*_CO_: transfer coefficient of the lung for carbon monoxide; LLN: lower limit of normal (<−1.645); TLC: total lung capacity. ^#^: p-values comparing high-risk and standard-risk patients were calculated using independent t-tests for continuous variables, and chi-squared or Fisher's exact tests for categorical variables. ^¶^: effect estimates compare the high-risk with the standard-risk group. For continuous outcomes, estimates are mean differences with 95% CIs from linear regression; for binary outcomes, estimates are absolute risk differences (percentage-point differences) with 95% CIs from binomial regression; positive values indicate higher means or prevalence in the high-risk group. ^+^: units of measurement: FVC, TLC, FRC and FEV_1_ in litres; FEV_1_/FVC as percentage; FEF_25–75%_ in L·s^−1^; *D*_LCO_ in mmol·(min^−1^·kPa^−1^); and *K*_CO_ in mmol·(min^−1^·kPa^−1^·L^−1^). ^§^: any impaired parameter was defined as having at least one abnormal pulmonary function parameter (z-score<–1.645) among the reported spirometry, body plethysmography or *D*_LCO_ indices. ^ƒ^: impairments were defined as: obstructive (FEV_1_/FVC<LLN and FVC<LLN), restrictive (TLC<LLN), mixed (FEV_1_/FVC<LLN with FVC≥LLN) and diffusion capacity impairment (*D*_LCO_<LLN).

### Parameters indicating possible restriction

The mean FVC z-score was −0.22 (95% CI −0.37 to −0.06) in standard-risk, only slightly below the population mean (z-score=0), while in high-risk survivors it was even lower (−0.91, −1.16 to −0.66). TLC showed a similar pattern, with z-scores of −0.17, −0.33 to −0.01 in the standard-risk and −0.54, −0.78 to −0.31 in the high-risk group. Compared with standard risk, high-risk participants had lower FVC (mean z-score difference −0.69, 95% CI −0.99 to −0.39) and TLC (−0.37, −0.66 to −0.08). Restrictive impairment (TLC<LLN) [22] was present in 8% and appeared more frequent in the high-risk (12%) than the standard-risk group (6%, p=0.138) (table 2). Most pulmonary function impairments were mild across all parameters, with few moderate or severe cases (supplementary table S3).

### Parameters indicating possible obstruction

The mean FEV_1_ z-score was within normal limits in the standard-risk (−0.10, −0.26 to 0.06) but reduced in the high-risk group (−0.70, −0.96 to −0.43). The FEV_1_/FVC ratio remained preserved in both groups (z-scores 0.24 and 0.41), and FEF_25–75%_ did not differ. Obstructive impairment (FEV_1_/FVC<LLN with FVC≥LLN) [22] was rare (4% overall), occurring slightly more often in the standard-risk group (6% *versus* 1%). Mixed impairment (FEV_1_/FVC<LLN and FVC<LLN) [22] was found in only two (1%) participants.

### Diffusion capacity parameters

*D*_LCO_ was normal in the standard-risk (mean z-score 0.32, 0.15 to 0.49), while the high-risk group showed slightly reduced values (−0.27, −0.72 to 0.18). Diffusion impairment (*D*_LCO_<LLN) [22] was found in 13% of high-risk compared with 4% of standard-risk survivors (p=0.027). Among those with impaired *D*_LCO_, *K*_CO_ was normal or low (<LLN in only three (2%)).

**FIGURE 1:**
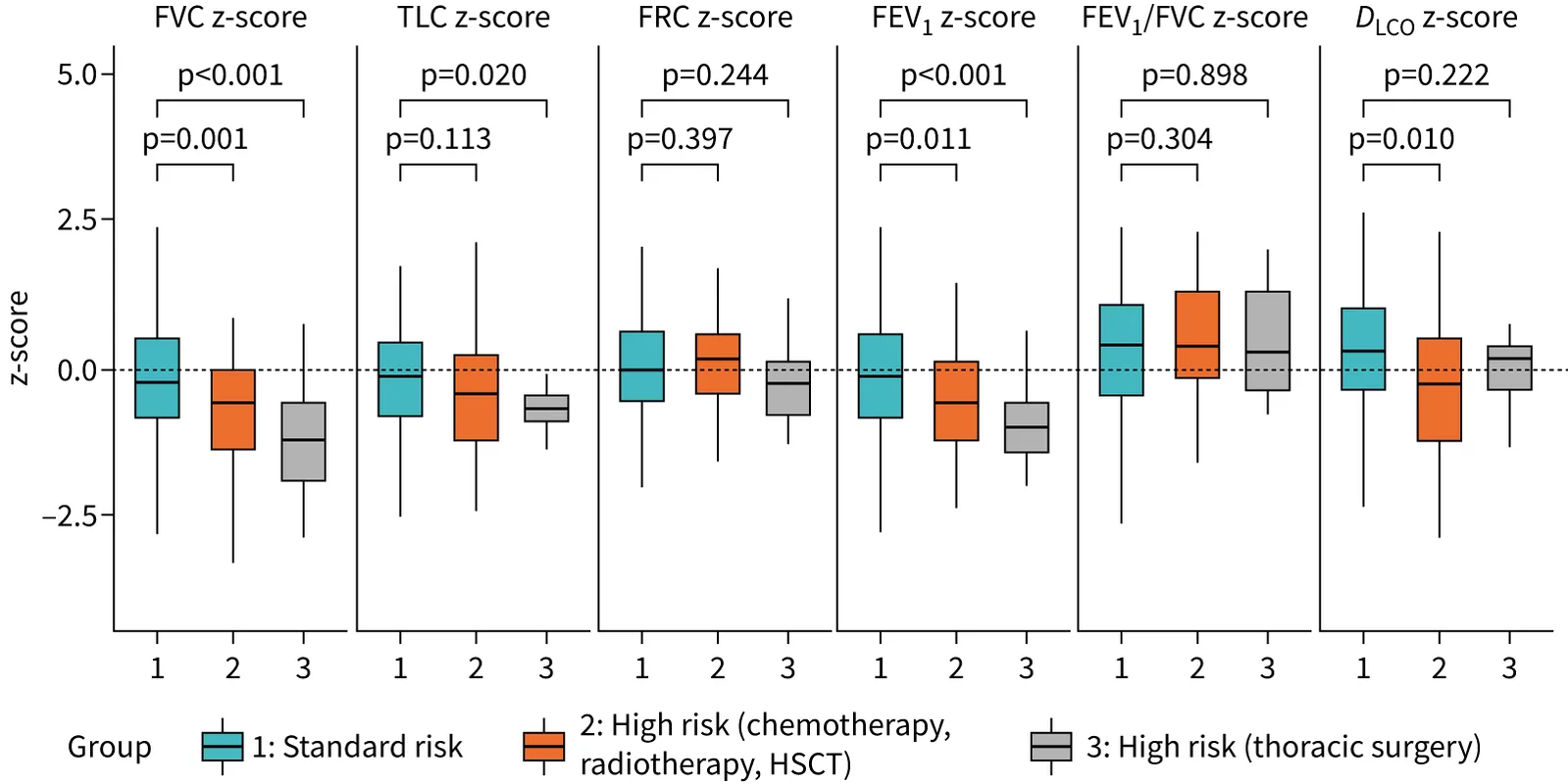
Pulmonary function outcomes in childhood cancer survivors compared across risk groups: standard risk (exposed to any systemic anticancer treatment), high risk (exposed to pulmotoxic chemotherapy, radiotherapy or haematopoietic stem cell transplantation (HSCT), or combinations of those treatments) and high risk who underwent thoracic surgery. Box plots show median z-scores and interquartile ranges (coloured), with the dashed line indicating z-score=0. p-values were calculated using two-sided independent t-tests comparing standard-risk *versus* high-risk groups. FVC: forced vital capacity; TLC: total lung capacity; FRC: functional residual capacity; FEV_1_: forced expiratory volume in 1 s.

Further stratification of the high-risk group based on thoracic surgery showed that restrictive changes were similarly frequent in both risk groups (12% and 11%), but TLC was more reduced after surgery (z-score −0.73 *versus* −0.45). Diffusion impairment was confined to those without thoracic surgery but with other high-risk treatments (18% *versus* 0%), reflected by lower mean *D*_LCO_ z-scores (−0.23 *versus* −0.01) (supplementary table S4 and figure 1).

### Association between treatment exposures and pulmonary function

In multivariable models, several treatment exposures were associated with impaired pulmonary function (table 3). Nitrosoureas were linked to reduced TLC z-scores (−1.37, 95% CI −2.18 to −0.57) and lower FEV_1_/FVC ratios (0.05, 0.01–0.10). Thoracic surgery was associated with reduced TLC z-scores (−0.53, −1.01 to −0.05), while pulmotoxic radiotherapy was associated with lower *D*_LCO_ z-scores (−0.63, −1.27 to −0.01). Nitrosoureas, thoracic surgery and HSCT were associated with both reduced FEV_1_ and FVC z-scores (supplementary table S5).

**TABLE 3:** Predictors of pulmonary function outcomes among childhood cancer survivors. Multivariable linear regression models showing associations of clinical characteristics and cancer treatments with indicators of obstruction (FEV_1_/FVC), restriction (TLC z-score) and diffusion impairment (*D*_LCO_ z-score)

Exposure	TLC z-score			FEV_1_/FVC z-score			*D*_LCO_ z-score		
	β	(95% CI)	p*-*value^#^	β	(95% CI)	p*-*value^#^	β	95%CI	p*-*value^#^
**Sex (ref. male)**	0.10	(−0.16–0.36)	0.452	0.14	(−0.13–0.41)	0.302	−0.22	(−0.58–0.14)	0.220
**Age at study (per year)**	−0.02	(−0.05–0.02)	0.347	−0.08	(−0.11–−0.04)	<0.001	−0.06	(−0.11–−0.01)	0.016
**Time since diagnosis (per year)**	−0.005	(−0.04–0.03)	0.772	0.06	(0.02–0.09)	0.002	0.04	(−0.01–0.09)	0.093
**Bleomycin (ref. no)**	0.33	(−1.02–1.68)	0.630	−0.14	(−1.59–1.30)	0.847	0.56	(−1.18–2.30)	0.529
**Busulfan (ref. no)**	0.61	(−0.15–1.37)	0.111	−0.25	(−1.03–0.52)	0.515	−0.04	(−1.02–0.94)	0.931
**Nitrosoureas^¶^ (ref. no)**	−1.37	(−2.18–−0.57)	0.001	0.83	(0.04–1.62)	0.040	−0.50	(−1.63–0.62)	0.379
**Thoracic surgery (ref. no)**	−0.53	(−1.01–−0.05)	0.032	0.05	(−0.45–0.56)	0.835	−0.05	(−0.64–0.73)	0.893
**Pulmotoxic radiotherapy (ref. no)**	−0.23	(−0.68–0.23)	0.306	0.29	(−0.20–0.78)	0.249	−0.63	(−1.27–−0.01)	0.045
**HSCT (ref. no)**	−0.33	(−1.01–0.35)	0.339	0.06	(−0.61–0.74)	0.851	−0.15	(−1.04–0.73)	0.731

*D*_LCO_: diffusing capacity of the lungs for carbon monoxide; FEV_1_: forced expiratory volume in 1 s; FVC, forced vital capacity; HSCT: haematopoietic stem cell transplantation; TLC: total lung capacity. ^#^: p-values are based on the t-statistics testing whether each regression coefficient differs significantly from zero in linear regression models. ^¶^: including carmustine and lomustine.

Among clinical covariates, older age at study was associated with lower FEV_1_, FVC and *D*_LCO_ z-scores. Sensitivity analyses showed similar results with additional adjustment for asthma (supplementary table S6); adjustment for study centre, smoking or BMI did not substantially change estimates. Asthma was associated with lower FEV_1_ and FEV_1_/FVC.

### Respiratory symptoms and their association with pulmonary function

32% (n=81) reported at least one respiratory symptom within the previous 12 months, with similar rates in standard- and high-risk groups (31% *versus* 34%; supplementary table S7).

**FIGURE 2:**
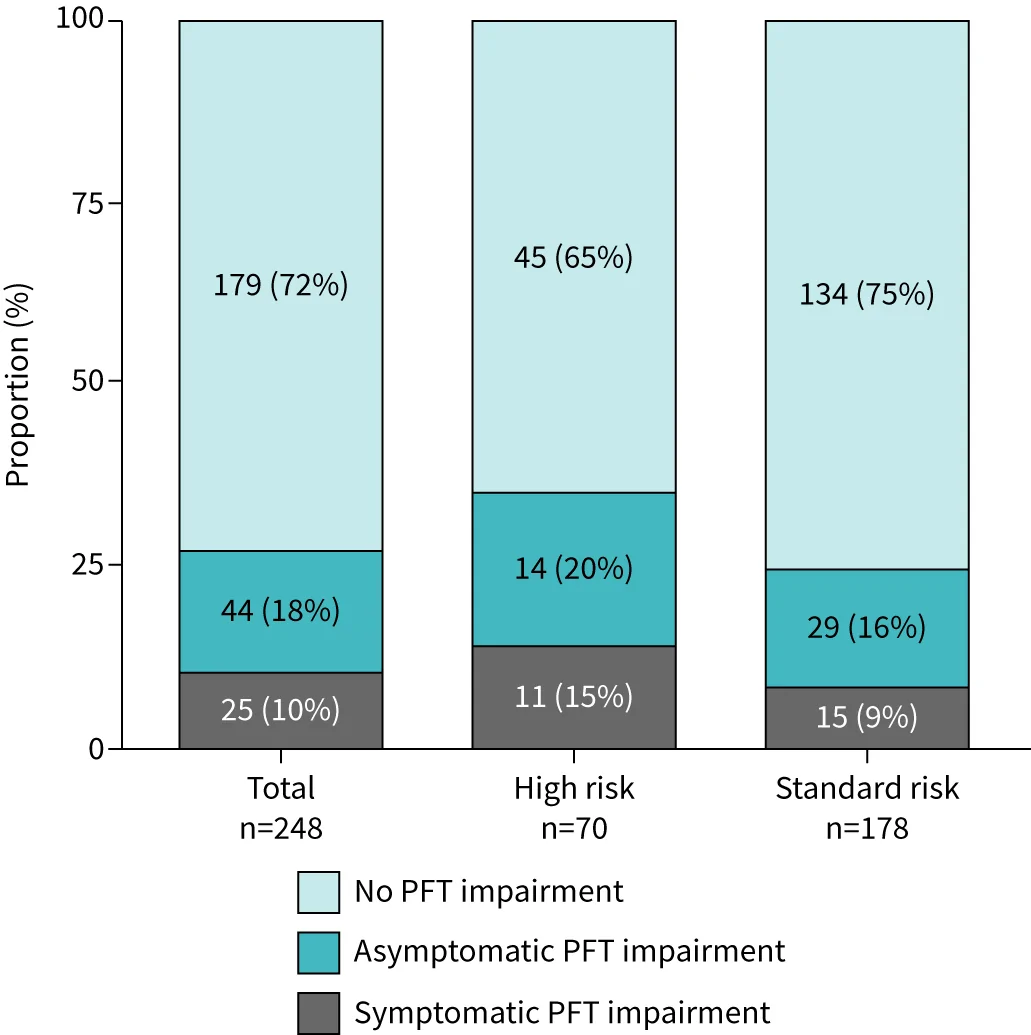
Proportion of childhood cancer survivors with no, asymptomatic and symptomatic pulmonary function test (PFT) impairment, stratified by treatment-related risk groups. The bars represent total population, high-risk and standard-risk groups, excluding three participants without questionnaire data. PFT impairment was defined as having at least one pathological z-score value of either forced expiratory volume in 1 s (FEV_1_), forced vital capacity (FVC), FEV_1_/FVC, forced expiratory flow at 25–75% of FVC (FEF_25–75%_), total lung capacity (TLC), functional residual capacity (FRC), diffusing capacity of the lungs for carbon monoxide (*D*_LCO_), transfer coefficient of the lung for carbon monoxide (*K*_CO_) below the lower limit of normal (z-score <−1.645). The lightest grey areas represent the proportion of participants with no PFT impairment. Reported symptoms were considered as occurring in the past 12 months and having had at least one of the following symptoms: at rest (wheeze, dyspnoea or cough), during exertion (wheeze, dyspnoea or cough), or chronic cough lasting for more than 4 weeks. Middle grey areas represent participants who did not report any of the symptoms but had a PFT impairment, while darkest areas represent participants who reported symptoms and had a PFT impairment.

We defined PFT impairment as any parameter (FEV_1_, FVC, FEV_1_/FVC, FEF_25–75%_, TLC, FRC, *D*_LCO_ or *K*_CO_) below the LLN. Among those with impairment (n=69), 36% reported at least one symptom, while 64% were asymptomatic. This pattern was similar across risk groups: 56% of high-risk and 67% of standard-risk survivors with impairment had no symptoms. Of those with symptoms (n=81), 31% had PFT impairment. There was no clear association between symptoms and PFT impairment (figure 2 and supplementary table S8).

## Discussion

In this prospective cohort of CCSs early after cancer diagnosis (median 7 years), PFT abnormalities were uncommon. Lung function in the standard-risk group was largely within normal ranges, whereas high-risk survivors more frequently showed mild reductions in parameters reflecting restriction and diffusion, with diffusion impairment being the most consistent abnormality. Nitrosoureas, thoracic surgery and HSCT were associated with abnormalities suggestive of restrictive or nonspecific ventilatory defects, while radiotherapy was linked to diffusion impairments. One-third reported respiratory symptoms, but over half of those with any pulmonary function impairment remained asymptomatic.

Restrictive and diffusion impairments were the most common patterns, affecting 8% and 7% of survivors, and occurred more frequently in high-risk survivors (12% and 13%). These rates exceed the 5% expected in a healthy population (defining LLN as −1.645 z-scores) and may indicate effects of lung-damaging treatments. However, group-level mean TLC and *D*_LCO_ z-scores remained above LLN, suggesting subclinical changes rather than overt pulmonary disease. Comparisons with prior studies are limited by inconsistent definitions, with many using % predicted thresholds not standardised for age, sex and height (supplementary table S9). We previously reported higher prevalences in Switzerland using GLI z-scores (z<−1.645). A retrospective nationwide study of survivors with the same high-risk profile, but on average 10 years post-treatment, found a prevalence of 22% restriction and 25% diffusion impairment [28], likely reflecting selection bias, as lung function data may have been more available for symptomatic CCSs or those treated more intensively. Similarly, in a retrospective study of CCSs post-HSCT, we found 32% restriction [29]. Even higher rates have been observed in adult high-risk survivors in the Netherlands (34% restriction, 42% diffusion impairment; z<−1.65) [30]. The lower prevalence observed here may reflect a shorter time since diagnosis. Additionally, not all observed patterns necessarily reflect intrinsic pulmonary pathology, as reduced *D*_LCO_ with reduced alveolar volume (*V*_A_) and preserved or increased *K*_CO_ can indicate incomplete alveolar expansion [31], and reduced TLC with preserved FRC can suggest reduced thoracic expansion rather than parenchymal disease [22].

Obstructive impairments were within the normal range expected for the general population (4% overall, 1% in the high-risk group), consistent with findings from young CCS cohorts (median age 16 years) showing 1–2% obstruction [28, 29]. Rates appear to remain low even with longer follow-up, such as 7% in our previous study of an adult high-risk CCS cohort (median age 30 years) [32]. These findings support that obstruction is not a predominant pattern in CCSs. The standard-risk group showed overall normal lung function, with <6% impairments for each outcome. With most studies focusing on high-risk groups, only a few in leukaemia survivors allow relevant comparison. In a 1998 Danish study (median age 16 years) 8% FEV_1_, 15% FVC and 11% TLC were below z-score −1.645 [33, 34]. Another study in leukaemia survivors (median age 15 years) using <80%predicted thresholds found 20–23% impairments for FEV_1_, FVC, TLC and *D*_LCO_. These higher rates may reflect older, more toxic treatment regimens [34]. Mean FEV_1_ and FVC z-scores in our standard-risk group (−0.10 and −0.22) were slightly higher than those reported in a Swiss general paediatric population (−0.50 and −0.43), indicating near-normal lung function and potential limitations of GLI reference values for Swiss children [35].

Several cancer treatments, including thoracic surgery, radiotherapy, HSCT, and nitrosoureas were associated with reduced pulmonary function, and associations were robust in sensitivity analyses. Thoracic surgery was associated with the most pronounced decreases in FEV_1_, FVC and TLC, likely reflecting reduced lung volume or chest wall compliance, as previously reported in survivors undergoing metastasectomy or structural resections [15, 36, 37]. Radiotherapy was associated with *D*_LCO_ impairment, but not with reduced TLC in our cohort. This differs from findings in adult survivors where both parameters were often affected [14, 15], and may reflect the shorter latency in our younger population. Nitrosoureas were independently associated with reduced TLC and higher FEV_1_/FVC, suggesting restrictive changes. These agents, known for their alkylating and carbamylating properties, have been implicated in delayed-onset pulmonary toxicity through interstitial damage and inflammation [7, 38]. Similarly, survivors who underwent HSCT had lower FEV_1_ and FVC, with preserved FEV_1_/FVC and no clear restriction, suggesting a nonspecific ventilatory defect possibly related to subtle parenchymal or chest wall changes [10, 39]. These findings highlight the importance of considering cumulative exposure profiles in surveillance, as patients with multiple risk factors may be especially vulnerable. Importantly, the expected association of obstructive impairments with asthma supports internal validity [40]. The association of older age at follow-up with lower FEV_1_, FVC and *D*_LCO_ may reflect cumulative damage or age-related decline.

Current IGHG guidelines recommend pulmonary function testing in symptomatic CCS, particularly those exposed to thoracic radiotherapy, surgery or allogeneic HSCT [8]. This approach reflects limited and inconsistent evidence linking treatment exposures to long-term pulmonary dysfunction in asymptomatic survivors. However, our findings suggest that relying on symptoms alone may miss a substantial proportion of cases. Among survivors with impaired pulmonary function, 64% were asymptomatic, a pattern consistent across risk groups and highlighting the frequency of subclinical dysfunction in this population. This may reflect the large pulmonary reserve and the tendency of children and adolescents to underperceive or inadequately communicate respiratory symptoms. Conversely, 32% of survivors reported respiratory symptoms, yet 69% of these had normal PFT results. Such symptoms may also result from transient conditions such as mild infections or allergies, deconditioning or cardiac conditions. These findings represent an important clinical implication. The marked mismatch between respiratory symptoms and objective pulmonary impairment indicates that symptom-based surveillance alone is insufficient, as symptom presence cannot reliably identify impairment and symptom absence does not exclude it. At the same time, effective interventions to reverse or delay pulmonary decline in asymptomatic survivors remain limited, and the extent to which surveillance alone improves long-term outcomes requires further study. Nevertheless, treatment-exposure-based monitoring appears more appropriate for risk stratification, preventive counselling and early recognition of progression, and provides a foundation for future interventional research.

This study is strengthened by prospective design with a high participation rate (90%), minimising selection bias and supporting the representativeness of our findings. The inclusion of a broad cohort of standard-risk survivors provided comprehensive lung function data, which was missing in most previous studies. Reporting pulmonary function as z-scores, rather than % predicted or categorical outcomes, accounts for age-, sex- and height-related variation, reduces misclassification in paediatric populations, and enables more accurate comparisons across studies and time points. Several limitations should be addressed. First, as in most survivorship studies, pre-treatment lung function data were not available. This limits our ability to distinguish treatment-related pulmonary dysfunction from pre-existing conditions. However, the median age at diagnosis was 5 years, and reliable results for PFTs are rarely feasible before this age due to insufficient cooperation and test complexity. Second, we lacked detailed data on radiotherapy dosimetry and lung volumes irradiated, which may have led to misclassification of pulmonary radiation exposure severity. Third, the number of survivors exposed to certain treatments was low, which limited statistical power to detect more nuanced associations. These exposure-specific associations should therefore be interpreted cautiously and considered exploratory, yet their direction remains consistent with prior evidence. Fourth, although we combined symptom questionnaires and resting pulmonary function outcomes, this analysis provides limited information on the clinical impact of observed impairments, particularly in asymptomatic survivors. Future studies should include chest imaging and cardiopulmonary exercise testing to clarify the structural and functional relevance of mild abnormalities and assess their impact on daily functioning and quality of life. Additional research is also needed to further explore the underlying pathophysiological mechanisms. Finally, long-term changes should be better characterised. We plan repeated pulmonary assessments through ongoing longitudinal follow-up and international pooling of data across prospective cohorts.

In this prospective cohort, lung function in standard-risk CCS was largely within normal limits up to 7 years post-treatment, with little evidence of impairments that would currently justify routine screening. In contrast, high-risk survivors, particularly those treated with nitrosoureas, thoracic surgery, HSCT or chest radiotherapy, showed reductions in pulmonary function that often remained asymptomatic. Although most abnormalities were mild, lung function values were consistently lower in high-risk survivors, and emerging longitudinal evidence suggests that such deficits may progress over time [41], supporting the need for continued monitoring. These findings reinforce that surveillance should be guided by treatment exposures rather than symptoms alone, as post-treatment pulmonary assessment can identify subclinical changes and provide an important baseline for long-term follow-up and care.

## Supplementary material

**Please note:** supplementary material is not edited by the Editorial Office, and is uploaded as it has been supplied by the author. Supplementary material 00500-2026.SUPPLEMENT2
