# Supplementary material for: Pulmonary function in young childhood cancer survivors: results from a prospective multicentre cohort
Source: ERJ Open Res. 2026 Sep 7;12(5):00500-2026. doi: 10.1183/23120541.00500-2026 (PMC13548452; doi:10.1183/23120541.00500-2026)
Supplement: Supplementary file 1 [file 00500-2026.SUPPLEMENT2.pdf]

Supplementary Materials

Pulmonary function in young childhood cancer survivors: results from a prospective multicentre cohort

Maša Žarković[1,2], Christina Schindera[1,3], Nicolas Waespe[1,4,5], Daniel Trachsel[6], Anne Mornand[7], Marc Ansari[5,8], Philipp Latzin[9], Claudia E Kuehni[1,4,9]

Affiliations

[1]Childhood Cancer Research Group, Institute of Social and Preventive Medicine, University of Bern, Switzerland

[2]Graduate School for Health Sciences, University of Bern, Switzerland

[3]Division of Paediatric Oncology/Haematology, University Children's Hospital Basel, University of Basel, Basel, Switzerland

[4]Division of Pediatric Hematology and Oncology, Department of Pediatrics, Inselspital, Bern University Hospital, University of Bern, Switzerland

[5]CANSEARCH Research Platform for Pediatric Oncology and Hematology, Department of Pediatrics, Gynecology and Obstetrics, Faculty of Medicine, University of Geneva, Switzerland

[6]Pediatric Intensive Care and Pulmonology, University Children's Hospital Basel, Basel, Switzerland

[7]Division of Pediatric Pulmonology, Department of Women, Child and Adolescent, University Hospital of Geneva, Switzerland

[8]Division of Pediatric Oncology and Hematology, Department of Women, Child and Adolescent, University Hospital of Geneva, Switzerland

[9]Division of Paediatric Respiratory Medicine and Allergology, Department of Paediatrics, Inselspital, University Hospital, University of Bern, Bern, Switzerland.

Corresponding author

Claudia Kuehni, Childhood Cancer Research Group, Institute of Social and Preventive Medicine, University of Bern, Mittelstrasse 43, 3012 Bern, Switzerland.

Email: claudia.kuehni@unibe.ch

Phone: +41 31 684 35 07

Supplementary Figure S1. Population tree of study participants of the SCCSS FollowUp – Pulmo study underwent a pulmonary function assessment.

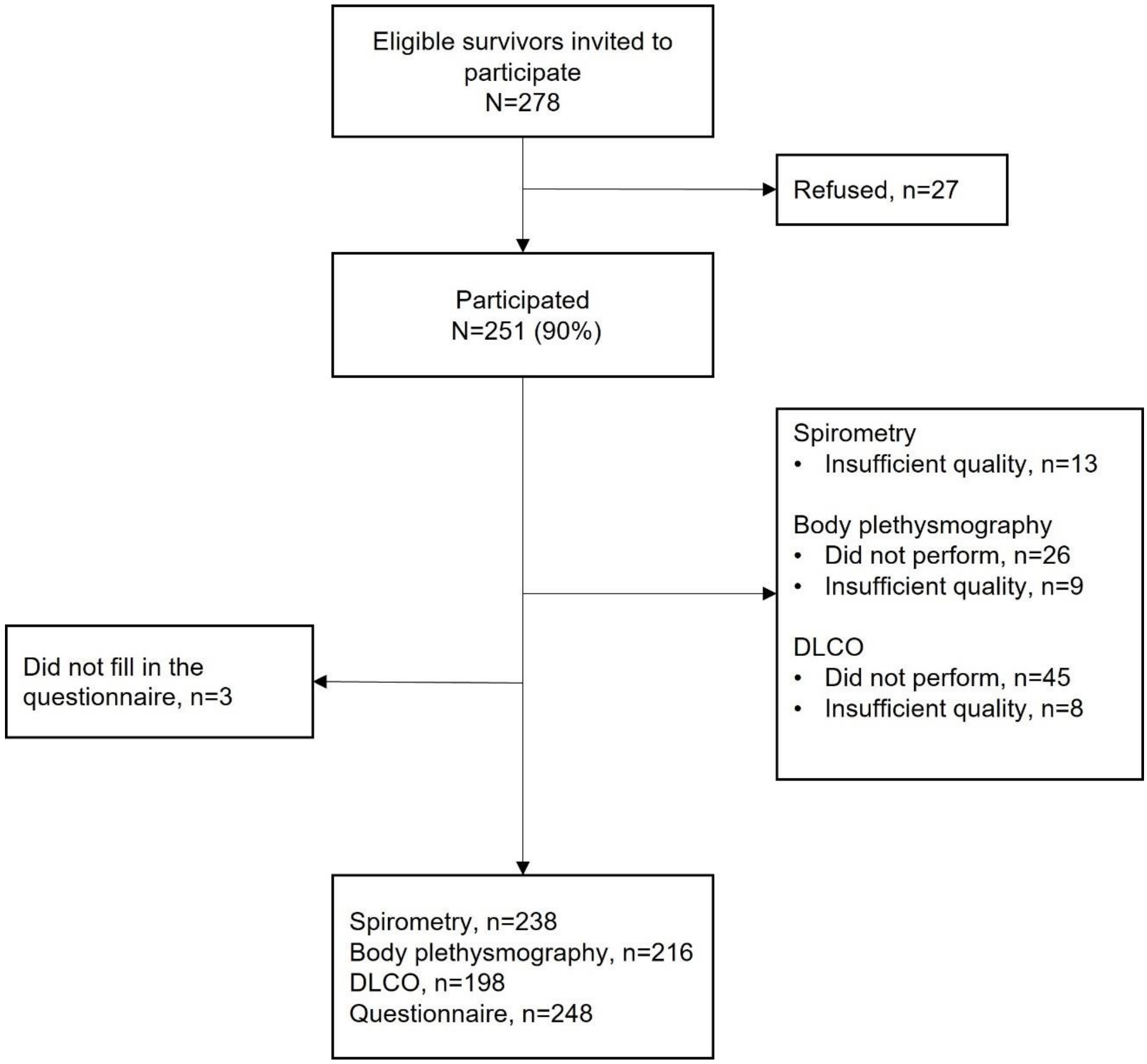

Spirometry was completed in all participants, with acceptable quality ranging from 86–99% across centres. Body plethysmography and DLCO were slightly less frequently performed (completion 77–95%), mainly due to test complexity and limited cooperation in younger children, but overall quality was generally acceptable, ranging from 90–96% for body plethysmography and 88–96% for DLCO across centres.
Abbreviations: DLCO, diffusing capacity of the lungs for carbon monoxide

Supplementary Table S1. Pulmonary function tests with main outcome parameters and questions on respiratory symptoms and smoking.

Pulmonary function tests	Unit	Details
**Spirometry**		
FEV_1	L	Forced expiratory volume in 1 second
FVC	L	Forced vital capacity
FEV_1/FVC	%	Ratio of forced expiratory volume in 1 second to forced vital capacity
FEF 25-75%	L/s	Forced expiratory flow at 25% to 75% of FVC
**Body plethysmography**		
TLC	L	Total lung capacity
FRC	L	Functional residual capacity
**DLCO**		
DLCO	mmol/(min*kPa)	Diffusing capacity of the lungs for carbon monoxide
KCO	mmol/(min*kPa*L)	Transfer coefficient of the lung for carbon monoxide (calculated as DLCO divided by alveolar volume [VA])

Questionnaire	Question
Respiratory symptoms at rest	Does your child / Do you sometimes have breathing difficulties without exertion? (yes/no)<br>If yes, which? [a]<br>• Wheeze<br>• Dyspnea<br>• Cough
Respiratory symptoms during exertion	Does your child / Do you sometimes have breathing difficulties on exertion? (yes/no)<br>If yes, which? [a]<br>• Wheeze<br>• Dyspnea<br>• Cough
Chronic cough	Has your child / Have you had cough in the last 12 months that lasted for more than 4 weeks? (yes/no)
Smoking (mother, father) [b]	Does someone smoke in the household (including normal cigarettes, e-cigarettes, or new devices such as iQOS)?<br>• Yes, only in the open<br>• Yes, also inside the home<br>• No, not anymore<br>• No, never
Smoking (adolescents) [c]	Have you ever smoked?<br>• No, never<br>• Yes, earlier, but I don’t smoke anymore<br>• Yes, I currently smoke, but not regularly<br>• Yes, I regularly smoke<br>How many hours per day are you exposed to passive smoking?<br>• X hours<br>• I don’t know<br>• I am not exposed to passive smoking

[a] Participants could report more than one symptom.

b Question asked in the questionnaire distributed to parents of survivors younger than 14 years

c Question asked in the questionnaire filled in by survivors older than 14 years

Supplementary Table S2. Pulmonary function test parameters, corresponding standard reference equations, and detailed specifications of measurement devices and software used at Bern, Basel, and Geneva study centers in the SCCSS FollowUp – Pulmo study.

PFT and parameters	Reference equations	Device, software; manufacturer (study center)
**Spirometry**		• MasterScreen Body plethysmograph, software version 3.20.3; CareFusion GmbH, Germany (Bern)<br>• Vyntus Body plethysmograph, software version 03.00; Vyaire Medical GmbH, Germany (Basel)<br>• Exp'Air spirometer, software version 2.01.03; MediSoft SA, Sorinnes, Belgium (Geneva)
FEV1 z-score	The global lung function 2012 equations:<br>• Quanjer et al, 2012	
FVC z-score		
FEV1/FVC z-score		
FEF25-75% z-score		
**Body plethysmography**		
TLC z-score	Official ERS technical standards:<br>• Hall et al, 2021	
FRC z-score		
**DLCO**		
DLCO z-score	Official ERS technical standards:<br>• Stanojevic et al, 2017<br>• Stanojevic et al, 2020	
KCO z-score		

Z-scores derived using the GLI calculator (https://gli-calculator.ersnet.org/), applying ethnicity-specific reference values based on participant-reported ethnicity.

Abbreviations: DLCO, diffusing capacity of the lungs for carbon monoxide; ERS, European Respiratory Society; FEF25–75%, forced expiratory flow at 25–75% of FVC; FeNO, fractional exhaled nitric oxide; FEV1, forced expiratory volume in 1 second; FRC, functional residual capacity; FVC, forced vital capacity; GLI, Global Lung Function Initiative; PFT, pulmonary function test; TLC, total lung capacity; z-score, standardized score based on reference equations.

Supplementary Table S3. Severity of pulmonary function impairments among childhood cancer survivors, reported by treatment-related risk groups.

	Total[a] N=251	Standard Risk[a] n=181	High Risk[a] n=70
**Restriction[b]**			
FVC z-score<LLN, n(%)	30 (13)	16 (9)	14 (22)
Mild	22 (9)	13 (7)	9 (14)
Moderate	8 (4)	3 (2)	5 (8)
Severe	0 (0)	0 (0)	0 (0)
TLC z-score<LLN, n(%)	16 (8)	9 (6)	7 (12)
Mild	13 (6)	6 (4)	7 (12)
Moderate	2 (1)	2 (1)	0 (0)
Severe	1 (<1)	1 (<1)	0 (0)
FRC z-score<LLN, n(%)	7 (3)	7 (4)	0 (0)
Mild	5 (2)	5 (3)	0 (0)
Moderate	2 (1)	2 (1)	0 (0)
Severe	0 (0)	0 (0)	0 (0)
**Obstruction[b]**			
FEV_1 z-score<LLN, n(%)	23 (10)	10 (6)	13 (21)
Mild	18 (8)	8 (5)	10 (16)
Moderate	5 (2)	2 (1)	3 (5)
Severe	0 (0)	0 (0)	0 (0)
FEV_1/FVC z-score<LLN, n(%)	13 (6)	12 (7)	1 (2)
Mild	11 (5)	11 (6)	0 (0)
Moderate	2 (1)	1 (<1)	1 (2)
Severe	0 (0)	0 (0)	0 (0)
$FEF_{25-75\%}$ z-score<LLN, n(%)	20 (8)	14 (8)	6 (10)
Mild	15 (6)	10 (6)	5 (8)
Moderate	5 (2)	4 (2)	1 (2)
Severe	0 (0)	0 (0)	0 (0)
**Diffusion impairment[b]**			
DLCO z-score<LLN, n(%)	13 (7)	6 (4)	7 (13)
Mild	10 (5)	6 (4)	4 (7)
Moderate	3 (2)	0 (0)	3 (6)
Severe	0 (0)	0 (0)	0 (0)
KCO z-score<LLN, n(%)	3 (2)	2 (1)	1 (2)
Mild	3 (2)	2 (1)	1 (2)
Moderate	0 (0)	0 (0)	0 (0)
Severe	0 (0)	0 (0)	0 (0)

[a] Spirometry, body plethysmography, and DLCO indices were not available for all participants. For each test result, the proportions were calculated based on the total number of tests which were performed and of good quality. For spirometry, there were a total of 238 (standard risk 175, high risk 63), for body plethysmography, a total of 216 tests (standard risk 156, high risk 60), and for body plethysmography, a total of 198 tests (standard risk 143, high risk 55).

[b] Mild, moderate, and severe impairment are classified according to 2021 ATS/ERS technical standard as z-scores for mild from -1.65 to -2.5, moderate from -2.51 to -4.0, and severe <-4.1 [1]

Abbreviations: DLCO, diffusing capacity of the lungs for carbon monoxide; $FEF_{25–75\%}$, forced expiratory flow at 25% to 75% of FVC; FEV_1, forced expiratory volume in one second; FEV_1/FVC, ratio of forced expiratory volume in one second to forced vital capacity; FRC, functional residual capacity; FVC, forced vital capacity; Hb, hemoglobin; KCO, transfer coefficient of the lung for carbon monoxide; LLN, lower limit of normal (<-1.645); TLC, total lung capacity.

Supplementary Table S4. Pulmonary function parameters among childhood cancer survivors, reported by treatment-related risk groups. Parameters include measures of restriction, obstruction, and diffusion capacity, with prevalence of impairment and group comparisons.

	**Standard Risk** n=181, 72%	**High Risk** (chemotherapy, radiotherapy, HSCT) n=50, 20%	***P* Value**[a]	**High Risk** (thoracic surgery) n=20, 8%	***P* Value**[b]
**Restriction**					
FVC, n(%)	175 (97)	44 (88)		19 (95)	
Mean (95%CI)	3.28 (3.08 – 3.47)	2.79 (2.48 – 3.10)	0.021	3.05 (2.46 – 3.64)	0.453
Z-score, mean (95%CI)	-0.22 (-0.38 – -0.64)	-0.79 (-1.08 – -0.49)	0.001	-1.20 (-1.68 – -0.72)	<0.001
Z-score<LLN, n (%)	16 (9)	8 (18)	0.086	6 (32)	0.003
TLC, n(%)	156 (86)	41 (82)		19 (95)	
Mean (95%CI)	4.50 (4.25 – 4.75)	3.80 (3.42 – 4.19)	0.009	4.24 (3.58 – 4.91)	0.498
Z-score, mean (95%CI)	-0.17 (-0.33 – -0.01)	-0.45 (-0.76 – -0.15)	0.113p	-0.73 (-1.03 – -0.43)	0.020
Z-score<LLN, n (%)	9 (6)	5 (12)	0.154	2 (11)	0.420
FRC, n(%)	156 (86)	41 (82)		19 (95)	
Mean (95%CI)	2.31 (2.17 – 2.45)	2.05 (1.83 – 2.27)	0.090	2.24 (1.87 – 2.60)	0.733
Z-score, mean (95%CI)	0.01 (-0.14 – 0.15)	0.14 (-0.13 – 0.41)	0.397	-0.25 (-0.54 – 0.05)	0.244
Z-score<LLN, n (%)	6 (4)	3 (7)	0.398	0 (0)	1.000
**Obstruction**					
FEV_1 , n(%)	175 (97)	44 (88)		19 (95)	
Mean (95%CI)	2.87 (2.71 – 3.03)	2.49 (2.23 – 2.76)	0.020	2.69 (2.22 – 3.17)	0.485
Z-score, mean (95%CI)	-0.10 (-0.26 – 0.06)	-0.57 (-0.90 – -0.24)	0.011	-0.99 (-1.47 – -0.51)	<0.001
Z-score<LLN, n (%)	10 (6)	8 (18)	0.007	5 (26)	0.001
FEV_1/FVC, n(%)	175 (97)	44 (88)		19 (95)	
Mean (95%CI)	0.89 (0.88 – 0.90)	0.90 (0.88 – 0.92)	0.199	0.90 (0.87 – 0.92)	0.493
Z-score, mean (95%CI)	0.24 (0.07 – 0.40)	0.43 (0.11 – 0.76)	0.304	0.36 (-0.03 – 0.74)	0.898
Z-score<LLN, n (%)	12 (7)	1 (2)	0.474	0 (0)	0.612
$FEF_{25-75\%}$, n(%)	175 (97)	44 (88)		19 (95)	
Mean (95%CI)	3.35 (3.16 – 3.53)	3.14 (2.80 – 3.48)	0.329	3.26 (2.75 – 3.76)	0.770
Z-score, mean (95%CI)	-0.08 (-0.24 – 0.08)	-0.02 (-0.38 – 0.34)	0.752	-0.36 (-0.84 – 0.11)	0.269
Z-score<LLN, n (%)	14 (8)	4 (9)	0.760	2 (11)	0.660
**Diffusion impairment**					
DLCO corrected for Hb, n(%)	143 (79)	38 (76)		17 (85)	
Mean (95%CI)	8.07 (7.62 – 8.52)	6.71 (5.86 – 7.56)	0.007	7.73 (6.34 – 9.12)	0.632
Z-score, mean (95%CI)	0.32 (0.15 – 0.49)	-0.23 (-0.76 – 0.30)	0.010	-0.01 (-0.41 – 0.40)	0.222
Z-score<LLN, n (%)	6 (4)	7 (18)	0.003	0 (0)	1.000
KCO corrected for Hb, n(%)	143 (79)	38 (76)		17 (85)	
Mean (95%CI)	1.90 (1.85 – 1.95)	1.96 (1.79 – 2.13)	0.342	2.03 (1.85 – 2.21)	0.080
Z-score, mean (95%CI)	0.44 (0.28 – 0.61)	0.58 (0.02 – 1.14)	0.510	0.75 (0.22 – 1.23)	0.232
Z-score<LLN, n (%)	2 (1)	1 (3)	0.509	0 (0)	1.000

[a] P values comparing standard-risk and high-risk (chemotherapy, radiotherapy, HSTC) groups calculated from independent t-tests for continuous and chi-squared or Fisher's exact tests for categorical variables.

[b] P values comparing standard-risk and high-risk (thoracic surgery) groups calculated from Student's t-test for continuous and chi-squared or Fisher's exact tests for categorical variables.

Abbreviations: CI, confidence interval; DLCO, diffusing capacity of the lungs for carbon monoxide; FEF25–75%, forced expiratory flow at 25% to 75% of FVC; FEV1, forced expiratory volume in one second; FEV1/FVC, ratio of forced expiratory volume in one second to forced vital capacity; FRC, functional residual capacity; FVC, forced vital capacity; Hb, hemoglobin; KCO, transfer coefficient of the lung for carbon monoxide; LLN, lower limit of normal (<-1.645); TLC, total lung capacity.

Supplementary Table S5. Predictors of lung function (FEV_1 and FVC z-scores) among childhood cancer survivors. Results from multivariable linear regression models examining clinical and treatment-related exposures.

**Exposure**	**FEV_1 z-score**			**FVC z-score**		
	**ß**	**(95% CI)**	***P* Value [a]**	**ß**	**(95% CI)**	***P* Value [a]**
Sex (ref: male)	0.15	(-0.11 – 0.42)	0.258	0.08	(-0.19 – 0.35)	0.516
Age at study (per year)	-0.08	(-0.12 – -0.05)	<0.001	-0.04	(-0.08 – -0.01)	0.015
Time since diagnosis (per year)	0.01	(-0.02 – 0.05)	0.421	-0.02	(-0.05 – 0.02)	0.307
Bleomycin (ref: no)	0.43	(-1.00 – 1.86)	0.551	0.53	(-0.88 – 1.94)	0.463
Busulfan (ref: no)	0.27	(-0.49 – 1.03)	0.483	0.41	(-0.34 – 1.16)	0.284
Nitrosoureas [b] (ref: no)	-0.71	(-1.49 – 0.08)	0.078	-1.09	(-1.87 – -0.32)	0.006
Thoracic surgery (ref: no)	-0.92	(-1.42 – -0.41)	<0.001	-0.97	(-1.46 – -0.47)	<0.001
Pulmotoxic radiotherapy (ref: no)	0.01	(-0.48 – 0.49)	0.981	-0.14	(-0.62 – 0.33)	0.551
HSCT (ref: no)	-0.80	(-1.47 – -0.13)	0.022	-0.84	(-1.50 – -0.18)	0.013

[a] P values are based on the *t*-statistics testing whether each regression coefficient differs significantly from zero in linear regression models.

[b] Including carmustine or lomustine.

Abbreviations: FEV_1, forced expiratory volume in 1 second; FEV_1/FVC, ratio of forced expiratory volume in 1 second to forced vital capacity; FVC, forced vital capacity; HSCT, haematopoietic stem cell transplantation.

Supplementary Table S6. Predictors of pulmonary function outcomes among childhood cancer survivors. Multivariable linear regression models showing associations of clinical characteristics and cancer treatments with FEV_1/FVC, TLC z-score, DLCO z-score, FEV1 z-score, and FVC z-score, adjusted for asthma.

Exposure	TLC z-score			FEV_1/FVC			DLCO z-score		
	ß	(95% CI)	*P* Value [a]	ß	(95% CI)	*P* Value [a]	ß	95%CI	*P* Value [a]
Sex (ref: male)	0.10	(-0.16 – 0.37)	0.436	0.03	(0.01 – 0.05)	<0.001	-0.22	(-0.57 – 0.14)	0.227
Age at study (per year)	-0.02	(-0.06 – 0.02)	0.301	-0.005	(-0.007 – -0.003)	<0.001	-0.07	(-0.12 – -0.02)	0.005
Time since diagnosis (per year)	-0.003	(-0.04 – 0.03)	0.875	0.003	(0.001 – 0.004)	0.025	0.05	(0.003 – 0.10)	0.039
Bleomycin (ref: no)	0.34	(-1.01 – 1.69)	0.623	-0.01	(-0.10 – 0.07)	0.746	0.60	(-1.13 – 2.32)	0.495
Busulfan (ref: no)	0.62	(-0.14 – 1.38)	0.111	-0.02	(-0.07 – 0.02)	0.283	-0.02	(-0.99 – 0.95)	0.969
Nitrosoureas [b] (ref: no)	-1.38	(-2.19 – -0.58)	0.001	0.06	(0.01 – 0.10)	0.021	-0.59	(-1.71 – 0.54)	0.303
Thoracic surgery (ref: no)	-0.52	(-1.00 – -0.04)	0.033	-0.001	(-0.03 – 0.03)	0.978	0.05	(-0.64 – 0.73)	0.893
Pulmotoxic radiotherapy (ref: no)	-0.24	(-0.71 – 0.22)	0.306	0.03	(0.002 – 0.06)	0.034	-0.68	(-1.31 – -0.04)	0.037
HSCT (ref: no)	-0.32	(-1.01 – 0.36)	0.356	-0.001	(-0.04 – 0.04)	0.975	-0.12	(-1.00 – 0.75)	0.783
Asthma (ref: no)	0.16	(-0.41 – 0.73)	0.584	-0.06	(-0.09 – -0.03)	<0.001	0.81	(-0.01 – 1.59)	0.062

Exposure	FEV_1 z-score			FVC z-score		
	ß	(95% CI)	*P* Value [a]	ß	(95% CI)	*P* Value [a]
Sex (ref: male)	0.13	(-0.14 – 0.39)	0.351	0.08	(-0.18 – 0.35)	0.540
Age at study (per year)	-0.08	(-0.11 – -0.04)	<0.001	-0.04	(-0.08 – -0.01)	0.022
Time since diagnosis (per year)	0.005	(-0.03 – 0.04)	0.793	-0.02	(-0.06 – 0.02)	0.281
Bleomycin (ref: no)	0.40	(-1.01 – 1.82)	0.574	0.52	(-0.89 – 1.93)	0.468
Busulfan (ref: no)	0.25	(-0.50 – 1.00)	0.509	0.41	(-0.35 – 1.16)	0.288
Nitrosoureas [b] (ref: no)	-0.69	(-1.47 – 0.09)	0.083	-1.09	(-1.87 – -0.31)	0.006
Thoracic surgery (ref: no)	-0.93	(-1.42 – -0.43)	<0.001	-0.97	(-1.47 – -0.47)	<0.001
Pulmotoxic radiotherapy (ref: no)	0.06	(-0.42 – 0.55)	0.798	-0.14	(-0.62 – 0.35)	0.579
HSCT (ref: no)	-0.84	(-1.51 – -0.18)	0.013	-0.85	(-1.51 – -0.18)	0.013
Asthma (ref: no)	-0.63	(-1.17 – -0.09)	0.022	-0.10	(-0.64 – 0.44)	0.710

[a] P values are based on the *t*-statistics testing whether each regression coefficient differs significantly from zero in linear regression models.

[b] Including carmustine and lomustine.

Abbreviations: CI, confidence interval; DLCO, diffusing capacity of the lungs for carbon monoxide; FEV_1, forced expiratory volume in 1 second; FEV_1/FVC, ratio of forced expiratory volume in 1 second to forced vital capacity; FVC, forced vital capacity; HSCT, haematopoietic stem cell transplantation; TLC, total lung capacity.

Supplementary Table S7. Prevalence and types of self-reported respiratory symptoms in childhood cancer survivors by treatment risk group

	**Total [a]** **N=248, 99%**	**Standard Risk [a]** **n=178, 72%**	**High Risk** **n=70, 28%**	***P* Value[b]**
Any respiratory symptom[c]	*81 (32)*	*57 (31)*	*24 (34)*	*0.671*
Respiratory symptoms at rest, n (%)[d]	16 (6)	13 (7)	3 (4)	0.368
Wheeze	2 (1)	2 (1)	0 (0)	0.519
Dyspnea	8 (3)	6 (3)	2 (3)	0.606
Cough	6 (2)	5 (3)	1 (1)	0.465
Respiratory symptoms during exertion, n (%)[d]	53 (21)	38 (21)	15 (21)	0.940
Wheeze	15 (6)	7 (4)	8 (11)	0.023
Dyspnea	29 (12)	21 (12)	8 (11)	0.969
Cough	29 (12)	21 (12)	8 (11)	0.969
Chronic cough (>4 weeks)	41 (17)	29 (16)	12 (18)	0.827

[a] Three participants did not fill in the questionnaire.

[b] P values comparing high-risk and standard-risk patients calculated from chi-squared or Fisher's exact tests.

[c] Defined as having reported at least one of the following symptoms (at rest, during exertion, or chronic cough)

[d] Defined as having reported at least one of the following symptoms (wheeze, dyspnea, cough). Participants could report more than one symptom.

Supplementary Table S8. Comparison of recent respiratory symptoms by pulmonary function impairment status in childhood cancer survivors, overall and by treatment-related risk groups

	**Total N=248, 99%**			**Standard risk N=178, 72%**			**High risk N=70, 28%**		
	PFT impairment [a]			PFT impairment [a]			PFT impairment [a]		
	Yes	No	*P*-value[b]	Yes	No	*P*-value[b]	Yes	No	*P*-value[b]
Symptoms [c], n (%)	25 (31)	56 (69)	0.457	15 (26)	42 (74)	0.735	10 (42)	14 (58)	0.453
No symptoms [c], n (%)	44 (26)	123 (74)		29 (24)	92 (76)		15 (33)	31 (67)	

[a] Pulmonary function test (PFT) impairment was defined as having at least one pathological value (z-score < -1.645) of either FEV1, FVC, FEV1/FVC, FEF25-75%, TLC, FRC, DLCO, KCO.

[b] P values calculated from chi-squared

[b] Defined as having reported at least one respiratory symptom occurring in the 12 months prior to pulmonary function testing and including: at rest (wheeze, dyspnea, cough), during exertion (wheeze, dyspnea, cough), or chronic cough lasting for more than 4 weeks.

Abbreviations: DLCO, diffusing capacity of the lungs for carbon monoxide; FEF25–75%, forced expiratory flow at 25% to 75% of FVC; FEV1, forced expiratory volume in one second; FEV1/FVC, ratio of forced expiratory volume in one second to forced vital capacity; FRC, functional residual capacity; FVC, forced vital capacity; KCO, transfer coefficient of the lung for carbon monoxide; TLC, total lung capacity.

Supplementary Table S9. Summary of studies on pulmonary function testing in childhood cancer survivors including the prevalence of abnormal indices.

	First author, year, country	Population	Definition of pulmonary function impairments	Prevalence of abnormal indices
1	Armenian, 2015, USA [2]	N=121, median age: 32 years<br>Inclusion criteria: 1) previous exposure to pulmonary-toxic chemotherapy (bleomycin, busulfan, nitrosoureas), and/or (2) chest radiation, and/or<br>(3) history of allogeneic HCT with cGVHD, and/or (4) pulmonary surgery (lobectomy, metastectomy, or wedge resection).	Obstructive lung disease (FEV1/FVC < 0.7 and FEV1 < 80% of predicted)<br>Restrictive lung disease (TLC < 75% and FEV1 ≥ 80% predicted)<br>Diffusion capacity abnormality (DLCOcorr < 75% predicted)	Obstructive: 6.6 %<br>Restrictive: 30.6 %<br>Diffusion capacity abnormality: 53.7 %
2	De, 2015, USA [3]	N=49, median age ~17years<br>Treatment-related inclusion criteria: no restriction for cancer diagnosis, radiotherapy involving the lung	Obstructive = FEV1/FVC < 80%pred or abnormal FEV1 or<br>FEF25–75%pred with normal lung volumes (i.e., normal TLC)<br>Restrictive = TLC < 77%pred<br>Hyperinflation = RV/TLC ratio > 28%<br>Diffusion capacity impairment = DLCOadj < 65%pred or<br>DLCOadj/VA <4 ml/mm/Hg/min/L	FVC %pred, n(%): 12 (24)<br>FEV1 %pred, n(%): 14 (29)<br>FEV1/FVC %pred, n(%): 7 (14)<br>FEF25–5% %pred, n(%): 10 (20)<br>RV %pred, n(%): 10 (21)<br>TLC %pred, n(%): 7 (15)<br>RV/TLC, n(%): 10 (21)_<br>Phase II N2 % N2/L, n(%): 11 (27)<br>DLCO adj %pred, n(%): 4 (9)<br>DLCO adj/VA ml/mmHg/min/L, n(%): 2 (5)<br><br>Obstructive lung disease: 12 (24%) patients<br>Restrictive lung disease: 7 (15%)<br>Hyperinflation: 10 (20%)<br>Diffusion defects: 6 (14%)
3	Denbo, 2014, USA [4]	N = 21, mean age: 35 years<br>Treatment-related inclusion criteria: diagnosis of osteosarcoma and pulmonary metastasectomy	Threshold for pathological parameters = FVC < 80% pred;<br>FEV1 < 80% pred; TLC <75% pred;<br>DLCOcorr < 75% pred<br>Obstructive = FEV1/FVC < 0·70<br>Restrictive = TLC < 75%pred	TLC: 28.6%<br>DLCOcorr: 47.4%<br>FVC: 40%<br>FEV1: 47.6%<br><br>Obstructive: 0(%)<br>Restrictive: 6 (28.6%)

4	Ginsberg, 2015, Canada [5]	N=273 age unclear (children)<br>Treatment-related inclusion criterion: bone marrow transplantation	Threshold for pathological parameters = z-score < -2 SD	Mean z-scores 5+ years post-transplant:<br>DLCO: -1.54<br>FEV1: -1.70<br>FVC: -1.68<br>TLC: -1.12
5	Green, 2015, USA [6]	N=260, age range 3-21 years<br>Treatment-related inclusion criterion: embryonal brain tumors	Threshold for pathological parameters = FEV1 < 80%pred,<br>FVC < 80%pred, DLCO < 75%pred, TLC < 75% pred	24 and 60 months follow up<br>FEV1: 34/170 (20%) and 32/109 (29%)<br>FVC: 46/172 (27%) and 30/108 (28%)<br>DLCO: 27/118 (23%) and 21/84 (25%)<br>TLC: 13/138 (9%) and 10/92 (11%)
6	Green, 2016, USA [7]	N=606, median age 34 years<br>Treatment-related inclusion criteria: no restriction for cancer diagnosis, at least one pulmonary toxic treatment modality (1) bleomycin, busulfan, BCNU, or CCNU; and/or (2) radiation therapy to the chest, whole lung, mediastinum, axilla, minimantle, mantle, extended mantle, total lymphoid irradiation, subtotal lymphoid irradiation, or total body irradiation; and/or (3) surgical procedures (pulmonary lobectomy, metastasectomy, or wedge resection), at least one PFT measurement	Threshold for pathological parameters = FEV1 < 80%pred,<br>FVC < 80%pred, FEV1/FVC < 0·7, TLC < 75%pred,<br>DLCOcorr < 75%pred	FEV1: 50.7%<br>FVC: 47.2%<br>TLC: 31.2%<br>DLCOcorr: 44.6%<br><br>Obstructive lung defects (FEV1/FVC < 0.7): 0.8%<br>Restrictive lung defects (TLC < 75%): 31.2%
7	Hoffmeister, 2006, USA [8]	N=215, median age 19 years<br>Treatment-related inclusion criterion: myeloablative HSCT	Threshold for pathological parameters = TLC < 80%, FVC,<br>FEV1, FEV1/FVC < 80%, DLCO < 70%	DLCO: 77/162 (43%)
8	Inaba, 2010, USA [9]	N=89, median age ~22years<br>Treatment-related inclusion criteria: allogeneic HSCT and available PFT before HSCT	Threshold for pathological parameters = FEV1, FVC, TLC,<br>DLCOcorr < 80%pred, FEF25-75 < 67%pred, RV and FRC > 120%pred. FEV1/FVC < 0.8. RV/TLC > 0·3.<br>Obstructive = FEV1/FVC < 0.8, FEV1 < 80%pred, FEF25–75 < 67%pred<br>Restrictive = FVC < 80%pred, TLC < 80%pred,	Post-transplant<br>FEV1/FVC: 22.5%<br>FEV1: 36%<br>FEF2575%: 49.4%<br>RV: 28.1%<br>RV/TLC: 38.2%<br>FRC: 15.7%<br>FVC: 39.3%<br>TLC: 43.8%<br>DLCOcorr: 64%

9	Jenney, 1995, UK [10]	N=69, mean age 14.6 years<br>Treatment-related inclusion criterion: acute leukaemia, at<br>least one PFT available	Threshold for pathological parameters = FEV1, FVC, RV, FRC,<br>ITGV, RAW, SGAW, TLC, DLCO analyzed as <85%pred and <<br>80%pred	Proportion of <85%pred and <80%pred<br>FEV1: 36% and 23%<br>FVC: 32% and 20%<br>RV: 32% and 25%<br>DLCO: 29% and 19%<br>KCO: 21% and 13%<br>TLC: 26% and 20%
10	Van Kalsbeek, 2024, Netherlands [11]	N=105, median age 36.5 years<br>Group B: pulmonary toxic treatment (bleomycin, busulfan, carmustine, lomustine,<br>radiotherapy involving (partial) lung or mediastinal tissue, including total body irradiation or spinal irradiation, or thoracic surgery.)	Diffusion impairment<br>(defined as DLCO below the lower limit of normal (LLN)), restrictive<br>pulmonary dysfunction (TLC < LLN), and obstructive pulmonary<br>dysfunction (FEV1/FVC < LLN)	Diffusion impairment: 34.3%<br>Restrictive: 41.9%<br>Obstructive: 7.6%
11	Kasteler, 2022, Switzerland [12]	N=183, median age 16.7 years<br>Treatment-related inclusion criteria: chemotherapeutics bleomycin, busulfan and nitrosureas<br>(lomustine and carmustine), thoracic radiotherapy, thoracic<br>surgery, and HSCT	Threshold for pathological parameters (FEV1, FVC, FEV1/FVC, TLC, DLCO) = z-score <-1.645<br>Obstructive = FEV1/FVC < 0.7 and FEV1 z-score <-1.645<br>Restrictive = TLC z-score <-1.645<br>Diffusion capacity impairment : DLCO < -1.645	Abnormal<br>FEV1: 33%<br>FVC: 38%<br>FEV1/FVC: 2%<br>TLC: 22%<br>DLCO: 25%<br><br>Obstructive: 1%<br>Restrictive: 22%<br>Diffusion impairment: 25%
12	Khan, 2020, USA [13]	N = 61, median age 24years<br>Treatment-related inclusion criteria: no restriction for cancer<br>diagnosis, radiotherapy to the lung	Obstructive = FVC z-score > −1.645, FEV1 z-score < −1.645,<br>FEV1/FVC ratio z-score < −1.645.<br>Restrictive = TLC z-score<br><−1.645. Hyperinflation = RV/TLC ratio z-score >+1.645. DLCO<br>z-score < −1·645	Diffusing: 57.1%<br>Restrictive abnormalities: 52.4%<br>Obstructive disease: 23.8%
13	Leung, 2007, USA [14]	N = 155, median age 18.5 years<br>Treatment-related inclusion criteria: no restriction for cancer<br>diagnosis, allogeneic HSCT	Threshold for pathological parameters = FEV1/FVC<br>< 85%pred, TLC and DLCO < 80% pred	FEV1/FVC: 27%
14	Madanat-Hujuoja, 2014, Finland [15]	N = 51 (1993–2005)<br>Treatment-related inclusion criteria: no	Obstructive = FEV1 < 80% and FEV1/FVC < 80%	Restrictive abnormalities (22/30, 73%), and 8/30 (26%) had a restrictive

		restriction for cancer<br>diagnosis, allogeneic HSCT	Restrictive = FVC < 80% and FEV1/FVC > 80%	abnormality with an obstructive component
15	Marina, 1995, USA [16]	N = 37, median age 15 years<br>Treatment-related inclusion criteria:<br>Hodgkin lymphoma plus<br>mantle radiotherapy plus chemotherapy with COP and ABVD	Threshold for pathological parameters = FVC, TLC, DLCO,<br>DLCO/VA as %predicted; no cut-off values defined	Mean (SD) 2-year post diagnosis values:<br>FVC: 87 (3)<br>TLC: 90.6 (2.9)<br>DLCO: 81.7 (3.1)<br>DLCO/VA: 5.09 (17)
16	Mittal, 2021, India [17]	N = 154, median age 18 years<br>Treatment-related inclusion criteria:<br>Hodgkin lymphoma	Threshold for pathological parameters = FEV1, FVC, DLCO <<br>80%pred<br>Restrictive = FVC < 80%pred, FEV1/FVC ≥ 85<br>Mixed = FVC < 80%pred, FEV1/FVC < 85	Abnormal spirometry: 43.2%<br>Reduced DLCO: 42.0%
17	Mulder, 2011, Netherlands [18]	N = 193, median age 27.3 years<br>Treatment-related inclusion criteria: no restriction for cancer<br>diagnosis, at least one pulmonary toxic treatment modality<br>(bleomycin, pulmonary radiotherapy and/or pulmonary<br>surgery)	Obstructive = FEV1/VCmax < 0·70 and FEV1 < 80%pred<br>Restrictive = TLC < 75%pred or FVC < 75%pred with normal<br>FEV1/VCmax ratio if no TLC available<br>Diffusion capacity impairment = DLCO or KCO < 75%pred	Restrictive: 17.6%<br>Decreased carbon monoxide diffusion capacity: 39.9%<br>Obstructive: 2.1%
18	Myrdal, 2018, Norway [19]	N = 116, median age 29 years<br>Treatment-related inclusion criteria: acute lymphoblastic<br>leukaemia (chemotherapy only)	Threshold for pathological parameters = FVC, FEV1, FE1/FVC,<br>TLC, RV, DLCO, DLCO/VA; reported as absolute values and<br>percentage of predicted<br>Obstructive = FEV1/FVC < 0·7<br>Restrictive and DLCO impairment ≤ 80%pred	Restrictive: 3 (3%)<br>Obstructive: 7 (6%)<br>Gas diffusion impairment: 25 (22%)
19	Nysom, 1998, Denmark [20]	N = 94, median age 16.2 years<br>Treatment-related inclusion criteria: Acute lymphoblastic<br>leukaemia	Threshold for pathological parameters = FEV1, FVC, TLC,<br>DLCO as z-scores, abnormal if < -1.645	FEV1 : 8%<br>FVC : 15%<br>FEV1/FVC : 1%<br>TLC : 11%<br>DLCO : 13%
20	Nysom, 1998, Denmark [21]	N = 41, median age 21.3 years<br>Treatment-related inclusion criteria:<br>Hodgkin and non-<br>Hodgkin lymphoma	Threshold for pathological parameters = FEV1, FVC, TLC,<br>DLCO as z-scores, abnormal if < -1.645	FEV1: 27%<br>FVC: 27%<br>FEV1/FVC: 10%
21	Oancea, 2014, USA [22]	N = 433, median age ~35 years<br>Treatment-related inclusion criteria: no restriction for cancer	Obstructive = FEV1/FVC < 0.70<br>Restrictive = TLC < 75%pred	Only one former smoker and one individual who had<br>never smoked met the criterion for

		diagnosis, at least one pulmonary toxic treatment modality (pulmonary lobectomy, metastasectomy or wedge resection, bleomycin, busulfan, lomustine, carmustine, or radiotherapy to the chest, whole lung, mediastinum, axilla, mini-mantle, mantle, extended mantle, total lymphoid irradiation, subtotal lymphoid irradiation, or total body irradiation)		diagnosis of obstructive lung disease (FEV1/FVC < 0.70). Restrictive lung disease (TLC < 75%) was present in 25.3% of current smokers, 30.4% of former smokers, and 34.6% of those who had never smoked.
22	Oguz, 2007, Turkey [23]	N = 75, median age 13 years<br>Treatment-related inclusion criteria: Hodgkin and non-Hodgkin lymphoma<br>Group **I** : 23 patients treated with both chemotherapy and thoracic irradiation<br>Group **II** : 52 patients treated with chemotherapy and no thoracic irradiation.	Obstructive by FEV1, FVC, FEV1/FVC. Restrictive by TLC, RV, RV/TLC ratio. Diffusion capacity impairment by DLCO. No further information.	Obstructive 0%<br>Restrictive : Group **I** 2/23 (9%), Group **II** 3/52 (6%)<br>Diffusion capacity impairment : Group **I** 5/23 (22%), Group **II** 4/52 (8%)
23	Otth, 2022, Switzerland [24]	N = 74, median age 16 years<br>Treatment-related inclusion criteria: autologous or allogeneic HSCT	Threshold for pathological parameters = FEV1, FVC, MMEF, TLC, RV, DLCO as z-scores, abnormal if z-scores < −1.645	FEV1: 35%<br>FVC: 38%<br>FEV1FVC: 1%<br>MMEF: 7%<br>TLC: 32%<br>RV: 9%
24	Record, 2016, USA [25]	N = 143, pediatric patients >5 years old<br>Treatment-related inclusion criteria: no restriction for cancer diagnosis, at least one pulmonary toxic treatment modality (bleomycin, busulfan, carmustine, lomustine; radiation to the chest (mantle, mediastinal, whole lung fields), abdomen (whole abdomen, upper abdominal field), or TBI; or surgery to the chest or lung (lobectomy, wedge resection, or	Obstructive = FVC < 80%pred.; FEV1 < 80%pred or FEV1/FVC < 80%pred, or FEF25-75% < 68%pred<br>Restrictive = TLC < 80%pred<br>Hyperinflation = RV > 120%pred or RV/TLC > 28%pred;<br>Abnormal TLC, FVC, FEV1, FEV1/FVC <80%, FEF2575 <68%, RV>120%, RV/TLC>28%, DLCO/VA<4ml/mmHg/min/L	Hyperinflation: 41.3%<br>Obstructive: 25.9 %<br>Restrictive disease: 13.3 %<br><br>Abnormal<br>TLC: 11%<br>FVC: 25.2%<br>FEV1: 25.9%<br>FEV1/FVC: 4.9%<br>FEF25-75: 11.9%<br>RV: 53.5%<br>RV/TLC: 51.9%<br>DLCO/VA: 5.5%

		thoracotomy))		
25	Schindera, 2021, Switzerland [26]	N=46, median age 30 years<br>Treatment-related inclusion criteria: high-risk = pulmotoxic chemotherapy, chest radiation, thoracic surgery, and/or HSCT; standard-risk: other cancer therapies	Threshold for pathological parameters = FEV1, FVC, FEV1/FVC as z-scores, abnormal if z-scores < −1.645	Abnormal (total, high-, standard-risk)<br>FEV1: 13%, 33%, 0%<br>FVC: 16%, 33%, 4%<br>FEV1/FVC: 5%, 7%, 4%
26	Stone, 2020, USA [27]	N = 62, 6-21 years old<br>Treatment-related inclusion criteria: High-risk<br>neuroblastoma	Threshold for pathological parameters = FEV1, FVC, TLC,<br>DLCO < 80%pred<br>Obstructive = FEV1/FVC < 0·8<br>Restrictive = TLC < 80 %pred	Abnormal<br>FVC: 53%<br>FEV1: 47%<br>TLC: 42%<br>DLCO: 71%<br><br>Obstructive: 6%<br>Restrictive: 35%<br>Mixed obstructive and restrictive: 6%
27	Weiner, 2006, USA [28]	N = 30, mean age 12.1 years<br>Treatment-related inclusion criteria: no restriction to cancer<br>diagnosis, whole lung irradiation	FVC, FEV1, FEV1/FVC, TLC, DLCO, MIP, MEP<br>2. Each pulmonary function parameter was considered normal<br>if it was within two standard deviations of the mean<br>(−2<Z < 2)	Abnormal (z-score <-2.00)<br>FEV1 : 50%<br>FVC : 53%<br>TLC : 60%<br>DLCO : 81%
28	Wieringa, 2005, Netherlands [29]	N = 39, median age ~15 years<br>Treatment-related inclusion criteria: no restriction to cancer<br>diagnosis, allogeneic HSCT	Threshold for pathological parameters = FEV1, FVC, FRC, RV,<br>DLCO, pathological when < 80%predicted<br>Obstructive = FEV1/FVC < 80%pred<br>Restrictive = TLC < 80%pred<br>Diffusion capacity impairment = DLCO < 80%pred	Obstructive: 6%<br>Restrictive: 45%<br>Diffusion abnormality: 76%

Adapted from Otth M et al., "Recommendations for surveillance of pulmonary dysfunction among childhood, adolescent, and young adult cancer survivors: a report from the International Late Effects of Childhood Cancer Guideline Harmonization Group," EClinicalMedicine, 2024;69:102487, https://doi.org/10.1016/j.eclinm.2024.102487 [30]. Modifications include updating references and adding a column for the prevalence of pulmonary function impairments. Licensed under CC BY 4.0 (https://creativecommons.org/licenses/by/4.0/).

Abbreviations: DLCO, diffusion capacity of the lung for carbon monoxide; DLCO/VA, diffusion capacity of the lung for carbon monoxide per unit alveolar volume; DLCOadj, adjusted diffusion capacity of the lung for carbon monoxide; DLCOadj/VA, adjusted diffusion capacity per unit of alveolar volume; DLCOcorr, hemoglobin-corrected diffusion capacity of the lung for carbon monoxide; FEF25–75%, forced expiratory flow at 25–75% of pulmonary volume; FEV1, forced expiratory volume in 1 second; FEV1/FVC, ratio of forced expiratory volume in 1 second to forced vital capacity; FRC, functional residual capacity; FVC, forced vital capacity; ITGV, intrathoracic gas volume; KCO, carbon monoxide transfer coefficient; LLN, lower limit of normal; MEP, maximal expiratory pressure; MIP, maximal inspiratory pressure; MMEF, maximal mid-expiratory flow; N2, nitrogen; RAW, airway resistance; RV, residual volume; RV/TLC, ratio of residual volume to total lung capacity; SGAW, specific airway conductance; TLC, total lung capacity; VA, alveolar volume; VCmax, maximum vital capacity.

References

1. Stanojevic S, Kaminsky DA, Miller MR, Thompson B, Aliverti A, Barjaktarevic I, Cooper BG, Culver B, Derom E, Hall GL, Hallstrand TS, Leuppi JD, MacIntyre N, McCormack M, Rosenfeld M, Swenson ER. ERS/ATS technical standard on interpretive strategies for routine lung function tests. European Respiratory Journal , 2022 60.
2. Armenian SH, Landier W, Francisco L, Herrera C, Mills G, Siyahian A, Supab N, Wilson K, Wolfson JA, Horak D, Bhatia S. Long-term pulmonary function in survivors of childhood cancer. Journal of Clinical Oncology , 2015 33: 1592–1600.
3. De A, Kamath S, Wong K, Olch AJ, Malvar J, Sposto R, Mascarenhas L, Keens TG, Venkatramani R. Correlation of pulmonary function abnormalities with dose volume histograms in children treated with lung irradiation. Pediatr Pulmonol , 2015 50: 596–603.
4. Denbo JW, Zhu L, Srivastava D, Stokes DC, Srinivasan S, Hudson MM, Ness KK, Robison LL, Neel M, Rao B, Navid F, Davidoff AM, Green DM. Long-term pulmonary function after metastasectomy for childhood osteosarcoma: A report from the st Jude lifetime cohort study. J Am Coll Surg , 2014 219: 265–271.
5. Ginsberg JP, Aplenc R, McDonough J, Bethel JJ, Doyle J, Weiner DJ. Pre-transplant lung function is predictive of survival following pediatric bone marrow transplantation. Pediatr Blood Cancer , 2010 54: 454–460.
6. Green DM, Merchant TE, Billups CA, Stokes DC, Broniscer A, Bartels U, Chintagumpala M, Hassall TE, Gururangan S, McCowage GB, Heath JA, Cohn RJ, Fisher MJ, Srinivasan A, Robinson GW, Gajjar A. Pulmonary function after treatment for embryonal brain tumors on SJMB03 that included craniospinal irradiation. Int J Radiat Oncol Biol Phys , 2015 93: 47–53.
7. Green DM, Zhu L, Wang M, Ness KK, Krasin MJ, Bhakta NH, McCarville MB, Srinivasan S, Stokes DC, Srivastava D, Ojha R, Shelton K, Pui CH, Armstrong GT, Mulrooney DA, Metzger M, Spunt SL, Navid F, Davidoff AM, Rao BN, Robison LL, Hudson MM. Pulmonary function after treatment for childhood cancer a report from the st. jude lifetime cohort study (SJLIFE). Ann Am Thorac Soc , 2016 13: 1575–1585.
8. Hoffmeister PA, Madtes DK, Storer BE, Sanders JE. Pulmonary function in long-term survivors of pediatric hematopoietic cell transplantation. Pediatr Blood Cancer , 2006 47: 594–606.
9. Inaba H, Yang J, Pan J, Stokes DC, Krasin MJ, Srinivasan A, Hartford CM, Pui CH, Leung W. Pulmonary dysfunction in survivors of childhood hematologic malignancies after allogeneic hematopoietic stem cell transplantation. Cancer , 2010 116: 2020–2030.
10. Jenney MEM, Faragher EB, Jones PHM, Woodcock A. Lung function and exercise capacity in survivors of childhood leukaemia. Med Pediatr Oncol , 1995 24: 222–230.
11. van Kalsbeek RJ, Feijen EAM, Bresters D, Kremer LCM, Pluijm SMF, Asogwa OA, Dulmen-den Broeder E van, van den Heuvel-Eibrink MM, Janssens GO, Tissing WJ, Loonen JJ, Neggers SJCMM, van der Pal HJH, Ronckers CM, Teepen JC, de Vries ACH, Louwerens M, van der Heiden-van der Loo M, Prevaes SMPJ, Versluys AB. Cyclophosphamide is not associated with clinically relevant late pulmonary dysfunction in Dutch survivors of childhood cancer – The DCCSS-LATER 2 PULM sub-study. Respir Med , 2025 237: 107948.
12. Kasteler R, Otth M, Halbeisen FS, Mader L, Singer F, Rössler J, von der Weid NX, Ansari M, Kuehni CE. Longitudinal assessment of lung function in Swiss childhood cancer survivors—A multicenter cohort study. Pediatr Pulmonol , 2024 59: 169–180.
13. Khan F, Williams AM, Weiner DJ, Constine LS. Impact of Respiratory Developmental Stage on Sensitivity to Late Effects of Radiation in Pediatric Cancer Survivors. Adv Radiat Oncol , 2020 5: 426–433.
14. Leung W, Ahn H, Rose SR, Phipps S, Smith T, Gan K, O'Connor M, Hale GA, Kasow KA, Barfield RC, Madden RM, Pui CH. A prospective cohort study of late sequelae of pediatric allogeneic hematopoietic stem cell transplantation. Medicine , 2007 86: 215–224.

15. Madanat-Harjuoja LM, Valjento S, Vettenranta K, Kajosaari M, Dyba T, Taskinen M. Pulmonary function following allogeneic stem cell transplantation in childhood: A retrospective cohort study of 51 patients. Pediatr Transplant , 2014 18: 617–624.

16. Marina NM, Greenwald CA, Diane L Fairclough, Thompson EI, Wilimas JA, Mackert PW, Hudson MM, Stokes DC, Bozeman PM. Serial pulmonary function studies in children treated for newly diagnosed Hodgkin's disease with mantle radiotherapy plus cycles of cyclophosphamide, vincristine, and procarbazine alternating with cycles of doxorubicin, bleomycin, vinblastine, and dacarbazine. Cancer , 1995 75: 1706–1711.

17. Mittal A, Bhethanabhotla S, Ganguly S, Vishnubhatla S, Khadgawat R, Patel C, Mohan A, Biswas A, Bakhshi S. Late effects in pediatric Hodgkin lymphoma survivors after uniform treatment with ABVD with or without radiotherapy. Pediatr Blood Cancer , 2021 68.

18. Mulder RL, Thönissen NM, Van Der Pal HJH, Bresser P, Hanselaar W, Koning CCE, Oldenburger F, Heij HA, Caron HN, Kremer LCM. Pulmonary function impairment measured by pulmonary function tests in long-term survivors of childhood cancer. Thorax , 2011 66: 1065–1071.

19. Myrdal OH, Kanellopoulos A, Christensen JR, Ruud E, Edvardsen E, Kongerud J, Sikkeland LI, Lund MB. Risk factors for impaired pulmonary function and cardiorespiratory fitness in very long-term adult survivors of childhood acute lymphoblastic leukemia after treatment with chemotherapy only*. Acta Oncol (Madr) , 2018 57: 658–664.

20. Nysom K, Holm K, Olsen J, Hertz H, Hesse B. Pulmonary function after treatment for acute lymphoblastic leukaemia in childhood. Br J Cancer , 1998 78: 21–27.

21. Nysom K, Holm K, Hertz H, Hesse B. Risk factors for reduced pulmonary function after malignant lymphoma in childhood. Med Pediatr Oncol , 1998 30: 240–248.

22. Oancea SC, Gurney JG, Ness KK, Ojha RP, Tyc VL, Klosky JL, Srivastava D, Stokes DC, Robison LL, Hudson MM, Green DM. Cigarette smoking and pulmonary function in adult survivors of childhood cancer exposed to pulmonary-toxic therapy: Results from the st. Jude lifetime cohort study. Cancer Epidemiology Biomarkers and Prevention , 2014 23: 1938–1943.

23. Oguz A, Tayfun T, Citak EC, Karadeniz C, Tatlicioglu T, Boyunaga O, Bora H. Long-term pulmonary function in survivors of childhood Hodgkin disease and non-Hodgkin lymphoma. Pediatr Blood Cancer , 2007 49: 699–703.

24. Otth M, Yammine S, Usemann J, Latzin P, Mader L, Spycher B, Güngör T, Scheinemann K, Kuehni CE, Ansari M, Beck Popovic M, Bourquin JP, Brazzola P, Greiner J, Rössler J, Schilling F, Scheinemann K, von der Weid N. Longitudinal lung function in childhood cancer survivors after hematopoietic stem cell transplantation. Bone Marrow Transplant , 2022 57: 207–214.

25. Record E, Williamson R, Wasilewski-Masker K, Mertens AC, Meacham LR, Popler J. Analysis of Risk Factors for Abnormal Pulmonary Function in Pediatric Cancer Survivors. Pediatr Blood Cancer , 2016 63: 1264–1271.

26. Schindera C, Usemann J, Zuercher SJ, Jung R, Kasteler R, Frauchiger B, Naumann G, Rueegg CS, Latzin P, Kuehni CE, von der Weid NX. Pulmonary dysfunction after treatment for childhood cancer comparing multiple-breath washout with spirometry. Ann Am Thorac Soc , 2021 18: 281–289.

27. Stone A, Friedman DN, Kushner BH, Wolden S, Modak S, LaQuaglia MP, Costello J, Wu X, Cheung NK, Sklar CA. Assessment of pulmonary outcomes, exercise capacity, and longitudinal changes in lung function in pediatric survivors of high-risk neuroblastoma. Pediatr Blood Cancer , 2019 66.

28. Weiner DJ, Maity A, Carlson CA, Ginsberg JP. Pulmonary function abnormalities in children treated with whole lung irradiation. Pediatr Blood Cancer , 2006 46: 222–227.

29. Wieringa J, van Kralingen KW, Sont JK, Bresters D. Pulmonary function impairment in children following hematopoietic stem cell transplantation. Pediatr Blood Cancer , 2005 45: 318–323.

30. Otth M, Kasteler R, Mulder RL, Agrusa J, Armenian SH, Barnea D, Bergeron A, Bhatt NS, Bourke SJ, Constine LS, Goutaki M, Green DM, Hennewig U, Houdouin V, Hudson MM, Kremer L, Latzin P, Ng A,

Oeffinger KC, Schindera C, Skinner R, Sommer G, Srinivasan S, Stokes DC, Versluys B, Waespe N, Weiner DJ, Dietz AC, Kuehni CE. Recommendations for surveillance of pulmonary dysfunction among childhood, adolescent, and young adult cancer survivors: a report from the International Late Effects of Childhood Cancer Guideline Harmonization Group. EClinicalMedicine , 2024 69: 102487.
